# Global‐Scale Patterns of Host Transitions Suggest Contrasting Levels of Specialization Within *Rhizoctonia solani*

**DOI:** 10.1002/ece3.74254

**Published:** 2026-09-01

**Authors:** Xin Zhi Khoo, Robert M. Harveson, Teddy Garcia‐Aroca

**Affiliations:** ^1^ Department of Ecology & Evolutionary Biology University of California Irvine California USA; ^2^ Department of Plant Pathology University of Nebraska‐Lincoln Lincoln Nebraska USA; ^3^ Panhandle Research Extension Center University of Nebraska‐Lincoln Scottsbluff Nebraska USA

**Keywords:** ecological specialization, generalism‐specialization continuum, genetic structure, host association, host range, host switch, intraspecific diversity, phylogenetic signal

## Abstract

The ability of plant pathogens to infect multiple host species has substantial adverse effects on food security and ecosystem health. *Rhizoctonia solani* is a globally distributed, soil‐borne fungal pathogen capable of infecting a wide variety of economically important crops. Its broad distribution across geographic regions and host taxa is accompanied by high genetic diversity. Historically, the *R. solani* species complex has been classified into 14 anastomosis groups (AGs) based primarily on hyphal anastomosis compatibility. Despite being widely perceived as a generalist, *R. solani's* host range has not been studied in a comprehensive cross‐AG framework to evaluate global patterns of pathogen diversity and host associations. We analyzed a global dataset of 4119 *R. solani* rDNA‐ITS sequences collected over the past 70 years, including newly sampled taxa from Nebraska, and used a smaller concatenated ITS and TEF‐1α dataset comprising 162 *R. solani* sequences to assess whether broad phylogenetic patterns were consistent across markers. Phylogenetic reconstruction suggests that most AGs formed well‐supported clades that largely corresponded to reported AG designations. Host family‐level transition inference suggests that generalist AGs may experience more transient host associations, while specialist lineages show stronger host‐associated genetic differentiation that contributes to the observed genetic structure. Our ancestral host‐state reconstruction analysis further suggests that the AG‐3 lineage likely originated from an ancestor with a broader host range and subsequently became predominantly associated with Solanaceae, with no inferred reversal in the sampled dataset. These findings suggest that host association and host‐transition patterns are important features of the generalism‐specialization continuum and contribute to population structure in a globally distributed fungal pathogen.

## Graphical abstract

We combined a global rDNA‐ITS dataset with TEF‐1α sequences and newly sequenced isolates to examine phylogenetic signal, host associations, and population structure in the soil‐borne pathogen *Rhizoctonia solani*. Most anastomosis groups formed well‐supported clades, and host‐associated patterns corresponded more strongly with genetic structure than geographic origin. Together, these results suggest that *R. solani* a generalism–specialization continuum, with some lineages showing broader host associations and others exhibiting stronger host‐associated genetic differentiation.

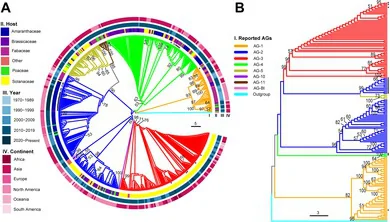

## 1 Introduction

*Rhizoctonia solani* is widely recognized as a species complex comprising genetically distinct lineages with diverse life histories (Anderson 1982). Collectively, lineages within *R. solani* infect more than 200 economically important plant species. Common diseases such as damping‐off, root rot, and sheath blight caused by *R. solani* affect numerous agricultural and horticultural plants worldwide, including cabbage, soybean, and rice (Yang 1999; Wang et al. 2015; Saygi et al. 2020). Traditionally, strains of *R. solani* have been recognized based on their compatibility in hyphal fusion reactions (anastomosis); which has led to the description of 14 anastomosis groups (AGs), including AG‐1 through AG‐13 and the bridging group, AG‐BI based on the ability of *R. solani* to fuse or anastomose their mycelia (Ogoshi 1987; Parmeter et al. 1969; Gónzalez et al. 2016). Some AG groups, like AG‐4, exhibit versatile pathogenicity, causing seedling and head rot diseases in various crops such as soybean, broccoli, tomato, melon, and cabbage (Chela Fenille et al. 2002; Muyolo et al. 1993a; Kuramae et al. 2003; Budge et al. 2009). AG‐1 is more host‐restricted, being primarily associated with sheath blight in cereal crops (Guleria et al. 2007; Vidhyasekaran et al. 1997; Feng et al. 2022), although occasional infections in Fabaceous hosts have also been reported (Rodriguez‐Herrera et al. 2022; Abbas et al. 2022). AG‐3 tends to infect a narrower range of hosts, showing a strong association with Solanaceae such as potato and tobacco (Watanabe and Matsuda 1966; Richter and Schneider 1953; Kuninaga et al. 2000; Woodhall et al. 2007). These patterns reflect a continuum of host associations across AG groups, indicating that *R. solani* comprises lineages with varying degrees of host range.

Many studies have focused on characterizing interactions between a single AG and a single host (Hane et al. 2014; Ghosh et al. 2018; Zheng et al. 2013), or at most a few AGs on a restricted range of hosts (Muyolo et al. 1993b; Akber et al. 2023). While these efforts provide valuable insights into specific host‐pathogen relationships, the narrow scope limits our understanding of broader host association patterns and the evolutionary forces shaping host range across the full spectrum of *R. solani* AGs. One way to contextualize this gap is through the concept of host switching, defined as the ability of pathogens to sustain infection and spread to novel host populations (Hafner et al. 1994; Gilbert and Webb 2007; De Vienne et al. 2013). The evolutionary outcomes of host‐switching events, however, vary widely across systems (Kamiya et al. 2014; De Meeûs et al. 1998). Empirical evidence from *Sclerotiniaceae* fungi suggests that repeated host jumps, combined with low speciation rates, have enabled generalist pathogens such as *Botrytis cinerea* and *Sclerotinia sclerotiorum* to evolve and maintain broad host ranges (Navaud et al. 2018). In the lichen‐parasitic fungus *Biatoropsis usnearum* system, host‐switching events play a different role by driving diversification, with each transition often leading to host‐specific adaptation and reproductive isolation (Millanes et al. 2014). A similar pattern of evolutionary flexibility is evident in wood‐decay fungi: white‐rot lineages tend to shift toward angiosperm specialization, whereas brown‐rot fungi frequently transition from gymnosperm specialists back to generalists (Krah et al. 2018). Given the ecological context of host ranges and global distribution of *R. solani* , it remains unclear whether host‐switching events promote generalism, specialization, or a mixture of both in this species complex.

Thus, this paper investigates whether host and lifestyle transitions among *R. solani* AGs occur randomly or follow directional patterns, either toward broader generalism or increased specialization. Directional shifts could reflect underlying evolutionary processes that contribute to the emergence of host‐restricted lineages, such as AG‐3. We predict that genetic relationships inferred from ribosomal DNA sequences will be concordant with traditional AG classifications. Given the well‐characterized host ranges of many AGs, we propose that host‐switching rates can be inferred from ribosomal DNA‐based phylogenies. Compared to conventional generalist populations, we anticipate that host‐specialized populations will show weaker genetic differentiation overall but a more pronounced correspondence between host association and population structure. To test these hypotheses, we combined phylogenetic reconstruction, Markov‐jump inference of host and lifestyle transitions, and analyses of population genetic structure. Understanding how host association and host switch shape adaptation and diversity in *R. solani* can improve predictions of future host‐switching events and guide strategies for disease management targeting certain *R. solani* lineages. To our knowledge, this study represents the first global‐scale cross‐AG phylogenetic and population structure analysis of host range evolution and genetic differentiation within *R. solani* .

## 2 Materials and Methods

### 2.1 Collection of *R. solani* Strains From Different Hosts

Symptomatic sugar beet and potato plants were collected in 2023 in Nebraska. The infected tubers and leaves were rinsed under slow‐running tap water to eliminate rhizosphere soil and debris. Infected tissues were dissected and sanitized with 70% ethanol for 3 min, rinsed with double deionized water (ddH_2_O) for 30 s, incubated in a 0.83% sodium hypochlorite (NaOCl) solution (CLOROXPRO, Oakland, CA) for 1 min, and rinsed again with ddH_2_O for another 30 s using a 3″ stainless steel tea infuser. The sterilized tuber pieces were placed on water agar (WA) amended with antibiotics 50 mg/L streptomycin sulfate (IBI Scientific, Dubuque, IA) and of 20 mg/L chloramphenicol (G‐Biosciences, St. Louis, MO) for initial isolation. Subculturing was performed to obtain pure cultures in half‐strength potato dextrose agar (HPDA) plates for fungal isolation. Thirty *R. solani* isolates were obtained from these recent sample collections. From previous collections, eight *R. solani* isolates that were initially collected from black‐eyed pea, cowpea, dry bean, and sugar beet, from 2001 to 2022, were revived from long term backups. We also included an isolate of *R. solani* that was isolated from tall fescue in 1992 (Giesler and Yuen 1998). A total of 38 *R. solani* isolates collected from six different host species across five counties in Nebraska were used in this study (Table S1).

### 2.2 DNA Extraction, PCR, and Sequencing

DNA was isolated using a modified DNeasy Plant Mini Kit (Qiagen, Hilden, Germany). Briefly, mycelia from 2‐week‐old pure cultures were scraped and added to a 1.5‐mL Eppendorf safe‐lock tube containing 400 μL buffer AP1 from the DNeasy Plant Mini Kit and 0.38 g of 0.5 mm and 0.39 g of 2.3 mm zirconium beads. Fungal tissue was disrupted using the Disruptor Genie Cell Disruptor Homogenizer model SI‐D238 (Scientific industries Inc., Bohemia, NY) for 3 min. The rest of the DNA isolation was carried out following the manufacturer's instructions. DNA quality and concentration for each isolate were estimated using a Nano‐Drop lite spectrophotometer (Thermo Scientific, Madison, WI) and stored at −20°C. The nuclear ribosomal DNA (nrDNA) from the internal transcribed spacers (ITS) region was amplified via PCR in a thermal cycler (Bio‐Rad Laboratories, Hercules, CA) using the universal primer set ITS 4 (5′‐TCCTCCGCTTATTGATATGC‐3′) and ITS 5 (5′‐GGAAGTAAAAGTCGTAACAAGG‐3′) (White 1990). The translation elongation factor 1‐alpha (TEF‐1α) region of these isolates was amplified using the primer set EF1‐688F (5′‐CGGTCACTTGATCTACAAGTGC‐3′) and EF1‐1251R (5′‐CCTCGAACTCACCAGTACCG‐3′) (Alves et al. 2008). The PCR mixture consisted of 1 μL of the template DNA, 1.0 μM of the forward and reverse primers described above (Integrated DNA Technologies (IDT), Newark, NJ), and the 2X GoTaq Hot Start Green Master Mix (Promega Corporation, Fitchburg, WI) for a total reaction volume of 11.5 μL. The ITS region was amplified using the following PCR parameters: initial denaturation at 95°C for 10 min, followed by 35 cycles of 30 s at 95°C, 30 s at 55°C, and 45 s at 72°C, and a final extension at 72°C for 10 min. The amplification conditions for the TEF‐1α region followed the previously described conditions: initial denaturation at 95°C for 5 min, followed by 30 cycles of 30 s at 94°C, 45 s at 55°C, and 90 s at 72°C, and a final extension at 72°C for 10 min (Phillips et al. 2005). Amplicons were verified by gel electrophoresis on a 1.5% agarose gel with 2.5 μL SYBR safe DNA gel stain nucleic acid dye (Invitrogen by Thermo Fisher Scientific, Carlsbad, CA) in 1X TAE buffer at constant 70 V for 120 min. The amplified product was visualized with a digital gel imaging system Molecular Imager Gel Doc XR + Imaging System 1 (Bio‐Rad Laboratories, Hercules, CA).

The amplified PCR products were submitted to Psomagen Inc. (Rockville, MD) for Sanger sequencing. The resulting sequences were assembled on Geneious Prime v2023.2 (https://www.geneious.com), using a DeNovo assembly approach in a medium sensitivity/fast setting. New sequences were classified according to the representative sequence of the same AG using the basic local alignment search tool (BLAST) with a 98% similarity threshold (Altschul et al. 1990). All newly sequenced isolates were stored in 30% glycerol (MilliporeSigma, Burlington, MA) at −80°C in the Fungal Ecology Lab Culture Collection (FELCC).

### 2.3 Obtaining Publicly Available ITS and TEF‐1α Sequences

Both ITS and TEF‐1α sequences were obtained from the National Center for Biotechnology Information (NCBI) GenBank database (https://www.ncbi.nlm.nih.gov/genbank/). The keywords used to capture most sequences were “*Rhizoctonia solani* AND Internal transcribed spacer,” “*Thanatephorus cucumeris* AND Internal transcribed spacer.” A total of 8354 ITS sequences of *R. solani* were downloaded from NCBI in July 2023. The metadata, including strain (AG), host, geographical origin, and collection date associated with each accession obtained from NCBI were extracted using a combination of customized Python v3.10.12 scripts. The same acquisition and data‐mining approaches were applied for the TEF‐1α gene, and 428 sequences were obtained.

### 2.4 Building Datasets and Multiple Sequence Alignments

From all sequences retrieved from NCBI, accessions with unknown collection year, unknown host species, and unknown geographical location were excluded. As the expected full‐length ITS region in *R. solani* typically ranges from 600 to 700 bp (Kuninaga et al. 1997), accessions that have sequences shorter than 400 base pairs and longer than 850 base pairs were also removed to exclude likely partial or chimeric sequences. ITS sequences of 38 FELCC isolates were integrated into the dataset, bringing the total to 4119 isolates of *R. solani. Athelia rolfsii* (AY684917) was included as an outgroup, bringing the ITS dataset to 4120 sequences. *A. rolfsii* was selected as the outgroup because it has been established as a reliable reference for rooting trees and elucidating evolutionary relationships within the *Rhizoctonia* complex (Sharon et al. 2007; Muzhinji et al. 2015; Ohkura et al. 2009). This dataset is henceforth referred to as “full ITS dataset”. For clearer data visualization and detailed examination of evolutionary relationships, we employed stratified random sampling method to create 5 subsets based on reported AGs with 461 publicly available sequences, 38 FELCC isolates, and an outgroup, totaling 500 sequences per subset. Sequences were aligned using the Multiple Alignment Fast Fourier Transform (MAFFT) v7 (Katoh and Standley 2013) program. The full ITS dataset was aligned using the progressive FFT‐NS‐2 method (Katoh et al. 2002) with default parameters (‐‐6merpair ‐‐maxiterate 0 ‐‐reorder), while the generated subsets were aligned using the iterative refinement G‐INS‐i method (Katoh et al. 2005) with default parameters (‐‐globalpair ‐‐maxiterate 2 ‐‐retree 1 –reorder).

We aligned ITS and matching TEF‐1α sequences using MAFFT with the G‐INS‐i method. The nucleotide alignments of both genes were concatenated using a public FastaCon script (GitHub repository: https://github.com/Pas‐Kapli/FastaCon/tree/master). The concatenated dataset comprised 162 *R. solani* sequences, including 144 publicly available sequences and 18 sequences from the FELCC collection. *A. rolfsii* was also included as an outgroup, bringing the concatenated dataset to 163 sequences. The final datasets used in this study consisted of: (I) a full ITS dataset (Table S1), (II) five smaller subsets derived from the full ITS dataset (hereafter referred to as “ITS subsets”), and (III) a concatenated ITS+TEF‐1α dataset (Henceforth referred to as “concatenated dataset”; Table S2). To remove ambiguously aligned regions, we trimmed all alignments using ClipKIT v1.4.1 (Steenwyk et al. 2020) in “gappy” mode with a 0.9 gap threshold. The extent of trimming varied across datasets. The full ITS dataset retained 795 alignment positions after trimming, with 56.6% of sites removed due to gaps or ambiguities. For one of the ITS subsets used in phylogenetic analysis, 771 sites were retained, and 27.5% of positions were trimmed. For the concatenated dataset, the ITS and TEF‐1α regions were aligned and trimmed separately prior to concatenation. The final ITS alignment retained 694 of 790 sites, while the TEF‐1α alignment retained 1000 of 1213 sites.

### 2.5 Phylogenetic Analyses

Maximum likelihood (ML) analyses were performed using the Randomized Accelerated Maximum Likelihood Next Generation (RAxML—NG) v1.2.2 (Kozlov et al. 2019) program. We first determined the appropriate substitution model for our datasets using ModelTest‐NG (Darriba et al. 2019). Hasegawa‐Kishino‐Yano model with gamma‐distributed rate variation among sites (HKY+ G4) was selected for the full ITS dataset based on model testing results, which showed this substitutionary model was the best fit for the respective data according to Akaike Information Criterion (AIC) and Bayesian Information Criterion (BIC). Additionally, bootstrap support analyses on RAxML‐NG with 1, 000 bootstrap replicates were inferred and mapped to the ML tree. Trees were visualized in R v4.3.1 and RStudio v2024.04.1 (Team RC 2024) using the “ggtree” package (Yu et al. 2017) and customized scripts. The same process was applied to ITS subsets and the concatenated dataset.

### 2.6 Markov‐Jump Analyses

From the full ITS dataset, we generated 10 independently host‐family‐stratified subsampled datasets for the Markov‐jump analyses. Each dataset contained 200 sequences, with 40 sequences randomly selected from each of the five major host families (Amaranthaceae, Brassicaceae, Fabaceae, Poaceae, and Solanaceae). Only sequences with complete AG annotations were included. This was done to reduce computational time while maintaining balanced representation among major host families. These major host families were selected based on their relevance to the evolutionary and ecological dynamics of *R. solani* observed in the ML analyses. Henceforth referred to as “host‐family‐stratified subsamples”. We estimated Markov jumps among the five major host families and performed an asymmetric discrete state phylogenetic analysis on the Bayesian Evolutionary Analysis Sampling Trees (BEAST) v1.10.4 program (Drummond and Rambaut 2007). The Markov‐jump analysis helps reconstruct host‐switching events by estimating the posterior expectation of the number of host change events across the branches of the phylogeny (Minin and Suchard 2008). We focused the Markov‐jump analyses on identifying host‐switching events without attempting to estimate the precise timing of these transitions. An uncorrelated log‐normal model was employed to account for substitution rate variation across branches. Given the nature of our ITS datasets, we chose the constant population model as it does not require assumptions about historical population size fluctuations (Kingman 1982). This model allowed us to focus on the genetic differentiation and host switch within *R. solani* populations, without attempting to infer specific demographic events over time. We performed a Markov Chain Monte Carlo (MCMC) run with 2 independent chains, using 100 million generations and sampling every 100 generations. Individual chains were first inspected separately using Tracer v1.7.2 to compare posterior distributions and assess mixing before these chains were combined. After removing 25% burn‐in from each chain, post‐burn‐in log and tree files were combined using LogCombiner v1.10.4. We calculated effective sample size (ESS) values from the combined log files using LogAnalyser v1.10.4 for key parameters, including the joint posterior, prior, likelihood, root height, and tree length. Maximum clade credibility (MCC) trees were generated from post‐burn‐in tree files using TreeAnnotator v1.10.4. To avoid biased sampling that could lead to biased results, the host state labels associated with every sequence in these 10 host‐family‐stratified subsets were randomized while maintaining the overall host distribution and then subjected to the same set of analyses.

### 2.7 Discriminant Analysis of Principal Components

To investigate the evolutionary dynamics of host specialization and generalism, we extracted all sequences associated with the AG‐3 and the AG‐4 clades from the full ITS phylogeny. Sequences with a high proportion of undefined character states were identified and removed to avoid potential biases in subsequent analyses. This resulted in two AG‐specific datasets: an AG‐3 dataset comprising 914 sequences and an AG‐4 dataset comprising 1174 sequences. Separate sequence alignments were then generated for each AG‐specific dataset.

To evaluate population structure without imposing host or geographic signals, we first used “find.clusters()” to perform k‐means clustering on the genetic data. The optimal number of clusters was evaluated using BIC. Discriminant Analysis of Principal Components (DAPC) was then performed using the inferred genetic clusters to visualize relationships among individuals. This is because DAPC focuses on maximizing the difference between groups while minimizing the variability within each group (Jombart et al. 2010). Host and geographic metadata were subsequently used to interpret the distribution of individuals among inferred clusters. Additional DAPC visualizations grouped by host category or geographic origin were used to evaluate whether these metadata categories corresponded to the observed genetic structure. The DAPC analysis was performed in R using the “adegenet” and “ggplot2” packages (Jombart 2008; Wilkinson 2011).

### 2.8 Population Genetic Analyses for *R. solani*

Standard diversity indices such as nucleotide diversity (*π*), polymorphism (*S*), number of haplotypes (*H*), and haplotype diversity (*Hd*) of each population were determined using DnaSP v6.12.03 (Rozas et al. 2017) to measure the genetic variability within these two populations. Several statistical analyses were conducted on DnaSP v6.12.03 to detect deviations from neutral evolution and infer demographic events in these two AGs. The Tajima's *D* (Tajima 1989) test was employed to detect deviations from the neutral theory of molecular evolution, which assumes that mutations are selectively neutral and that the population is at equilibrium under mutation and genetic drift. This test compares the number of segregating sites to the average number of pairwise nucleotide differences, with significant departures from zero suggesting population size changes or selection. Fu and Li's D* and F* (Fu and Li 1993) were similarly applied to assess neutrality by comparing the number of rare variants (singletons) to the total number of mutations; significant values may indicate purifying selection, recent population growth, or other nonneutral processes. Fu's *F*s (Fu 1997) tested for population expansion by comparing the observed number of alleles to the number expected under neutrality, with significant negative values suggesting population expansion.

To quantify host‐associated population differentiation, we performed Analysis of Molecular Variance (AMOVA) and pairwise distance‐based *F*_ST_ analyses within AG‐3 and AG‐4 using the AG‐specific datasets. For AG‐3, populations were defined by host groups as potato, tobacco, other Solanaceae hosts (“Others”), and non‐Solanaceae hosts. AG‐4 populations were defined by host families such as Amaranthaceae, Brassicaceae, Fabaceae, Poaceae, Solanaceae, and “Other”, with the latter group pooling isolates from 26 less frequently represented host families. AMOVA was performed in R using the “pegas” package (Excoffier et al. 1992; Paradis 2010), and statistical significance was assessed using 999 permutations. Pairwise distance‐based *F*_ST_ was calculated from mean within‐ and between‐population genetic distances (Hudson et al. 1992), with significance assessed by permutation of population labels.

### 2.9 Haplotype Network Analysis

Unique haplotypes identified by DnaSP were then imported into PopART (Population Analysis with Reticulate Trees; Leigh and Bryant 2015) to build a haplotype network of AG‐3 and AG‐4 using a median joining (MJ) algorithm (Bandelt et al. 1999). The MJ network was chosen for its efficiency in constructing networks that represent the most parsimonious evolutionary pathways among haplotypes. Each haplotype was annotated with host group and continent traits independently to explore the potential geographical and ecological structure within the subpopulation of these two AGs.

## 3 Results

### 3.1 Phylogenetic Reconstruction of *Rhizoctonia solani*

**FIGURE 1:**
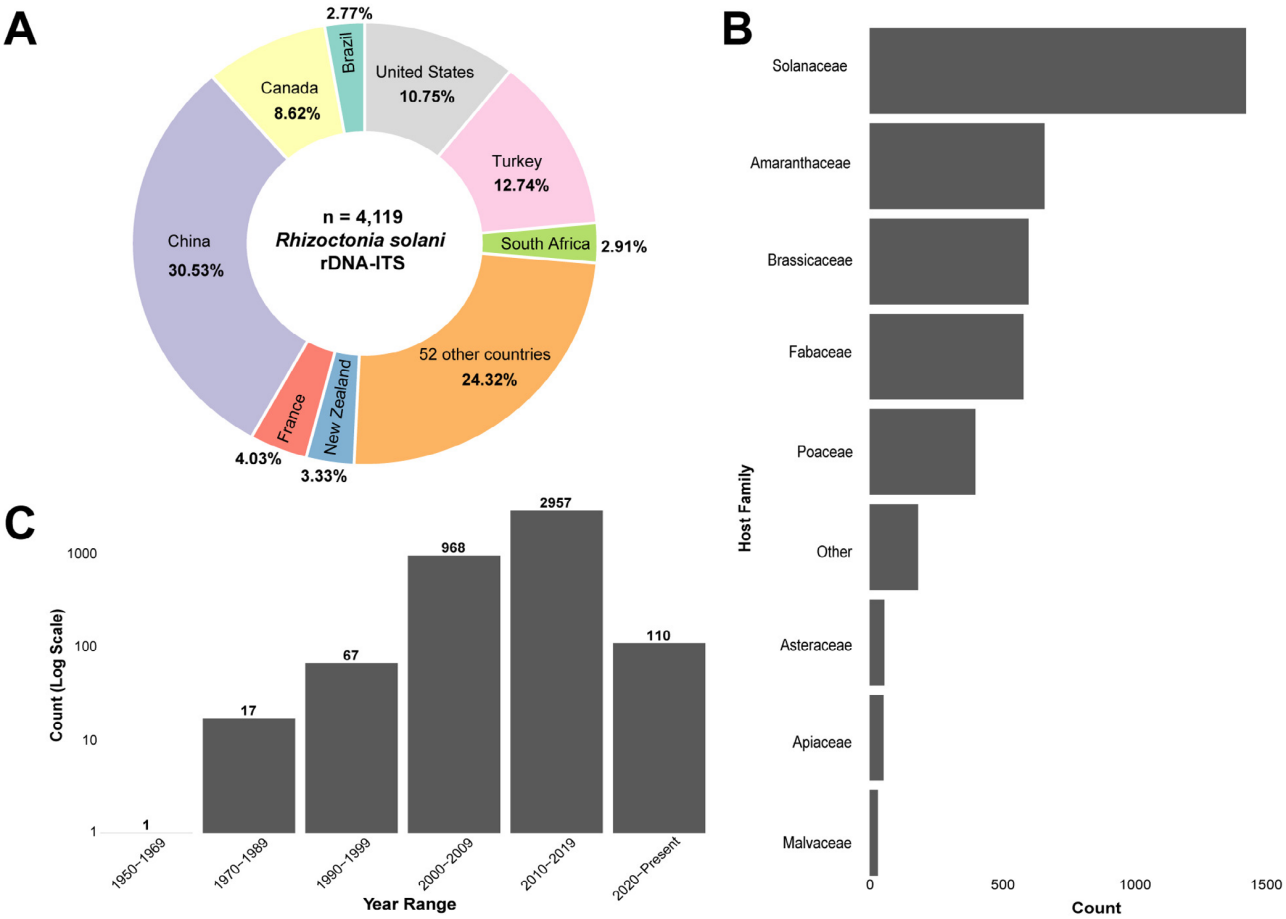
Global distribution of *Rhizoctonia solani*'s ribosomal DNA Internal Transcribed Spacers (ITS) sequence accessions available on NCBI GenBank (4119 ITS sequences as of July 2023), representing the global diversity, host range, and collection years of the dataset used in this study. (A) Percentage of ITS sequences obtained from 60 different countries. The “52 other countries” category includes countries that make up less than 1% of the sequences per country. (B) Bar plot summarizing the number of isolates collected from different host families. The ‘Other’ category includes 45 other less frequent host families for *R. solani* , ranging from Arecaceae to Zinguberaceae. A complete list of host families and ITS accessions used in this study can be found in the comprehensive metadata (Table S1). (C) Bar plot summarizing the number of isolates collected in different years. A logarithmic scale was applied for better visualization by emphasizing differences that are less noticeable on a linear scale.

**FIGURE 2:**
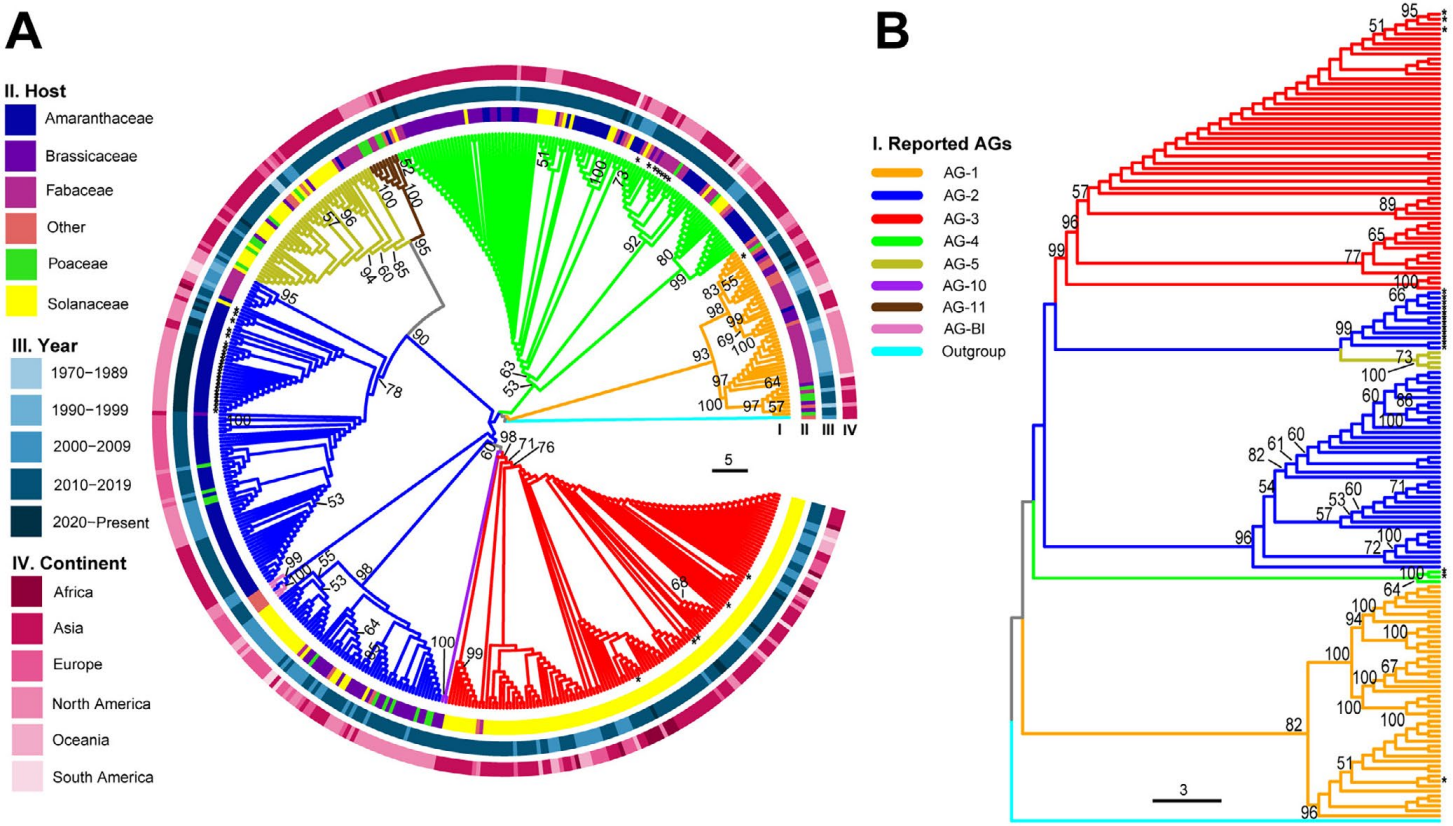
Phylogenetic reconstruction of *Rhizoctonia solani* from publicly available, published sequences and newly sequenced specimens (*). (A) Maximum likelihood (ML) phylogenetic tree inferred from a subset containing 500 rDNA‐ITS sequences (“ITS‐only phylogeny”), represented as a cladogram for visualization purposes. The tree demonstrates the genetic relationship among these sequences based and the internal nodes and branches are colored based on their reported anastomosis groups (AGs) on NCBI. The ML tree is further supplemented with additional layers representing host families, collection years, and geographic region (at the continental scale). (B) ML phylogenetic tree based on the concatenated dataset containing 162 rDNA‐ITS and TEF‐1α sequences (“concatenated ITS+TEF‐1α phylogeny”, Table S2). Internal nodes and branches are colored based on their reported anastomosis groups (AGs) on NCBI GenBank.

Our full ITS dataset was generated to represent the breadth of the known AG, host, geographic, and temporal diversity of *R. solani* at a global scale (Figure 1). Overall, the 4119 isolates formed nine distinct clades in the maximum‐likelihood (ML) phylogeny, corresponding to the reported *R. solani* AGs. The full ITS phylogeny (Figure S1) and the ITS‐only subset phylogenies (Figure 2A, Figure S1) were highly congruent. With the notable exceptions of AG‐2 and AG‐4, all AGs formed monophyletic clades with bootstrap support exceeding 70%. Our analyses revealed that AG‐2 can be divided into two distinct clades with high bootstrap support values, suggesting significant genetic differentiation within this group. This tree topology was also recovered in the concatenated ITS+TEF‐1α phylogeny (Figure 2B). Moreover, the existence of multiple subclades in several AGs highlights the genetic heterogeneity within each AG. For instance, three subclades can be observed in AG‐4. AG‐1, AG‐2, and AG‐3 also exhibit internal structures with multiple subclades, which correspond closely to the recognized subgroups within these AGs. Notably, AG‐BI was found clustering within one of the AG‐2 clades. This particular AG‐2 clade, which contains the majority of AG‐BI isolates, exhibits a closer phylogenetic relationship to AG‐3 and AG‐10. A few isolates were also found clustering within an AG clade that was different from their reported AGs in the full ITS phylogeny. Instances of isolates appearing in different AGs than the originally reported AG were observed specifically in AG‐2, AG‐4, AG‐5, AG‐11, and AG‐BI in both full and ITS‐only subset phylogenies.

We used ClipKIT gappy mode as the primary trimming strategy because the full ITS alignments contained many gap‐rich regions. To evaluate whether this trimming choice affected broad AG‐level phylogenetic relationships, we compared ML trees inferred from untrimmed MAFFT alignment and from alignments processed using ClipKIT gappy, ClipKIT kpic, ClipKIT smart‐gap, and Gblocks trimming strategies (Figure S2; Table S3). The major AG‐level relationships were generally recovered across trimming strategies, indicating that the broad phylogenetic placement of *R. solani* AGs was robust to alignment trimming, although some finer relationships and bootstrap support values varied among trimming choices.

### 3.2 Host Associations Among and Within AG Groups

We examined reported host associations among *R. solani* AGs on the ML phylogeny and found that most AGs infect a diverse array of host families, a pattern largely in agreement with the polyphagous nature of *R. solani* , and the reason why it is oftentimes referred to as a generalist. However, based on reported host metadata, AG‐3 and some subgroups within other AGs showed evidence of more restricted host associations. Notably, AG‐2 subclades associated with Amaranthaceae did not cluster closely with those associated with Brassicaceae (Figure 2A, Figure S2), suggesting potential host‐associated specialization within AG‐2 subgroups. In stark contrast to broader host‐family representation observed in AG‐2 and AG‐4, AG‐3 showed a strong association with Solanaceae, with nearly 97% of AG‐3 sequences in GenBank‐derived ITS dataset reported from hosts in this family (Figure S3).

### 3.3 Markov‐Jump Analyses Suggest Constrained Host Transitions Among Major Host Families

**FIGURE 3:**
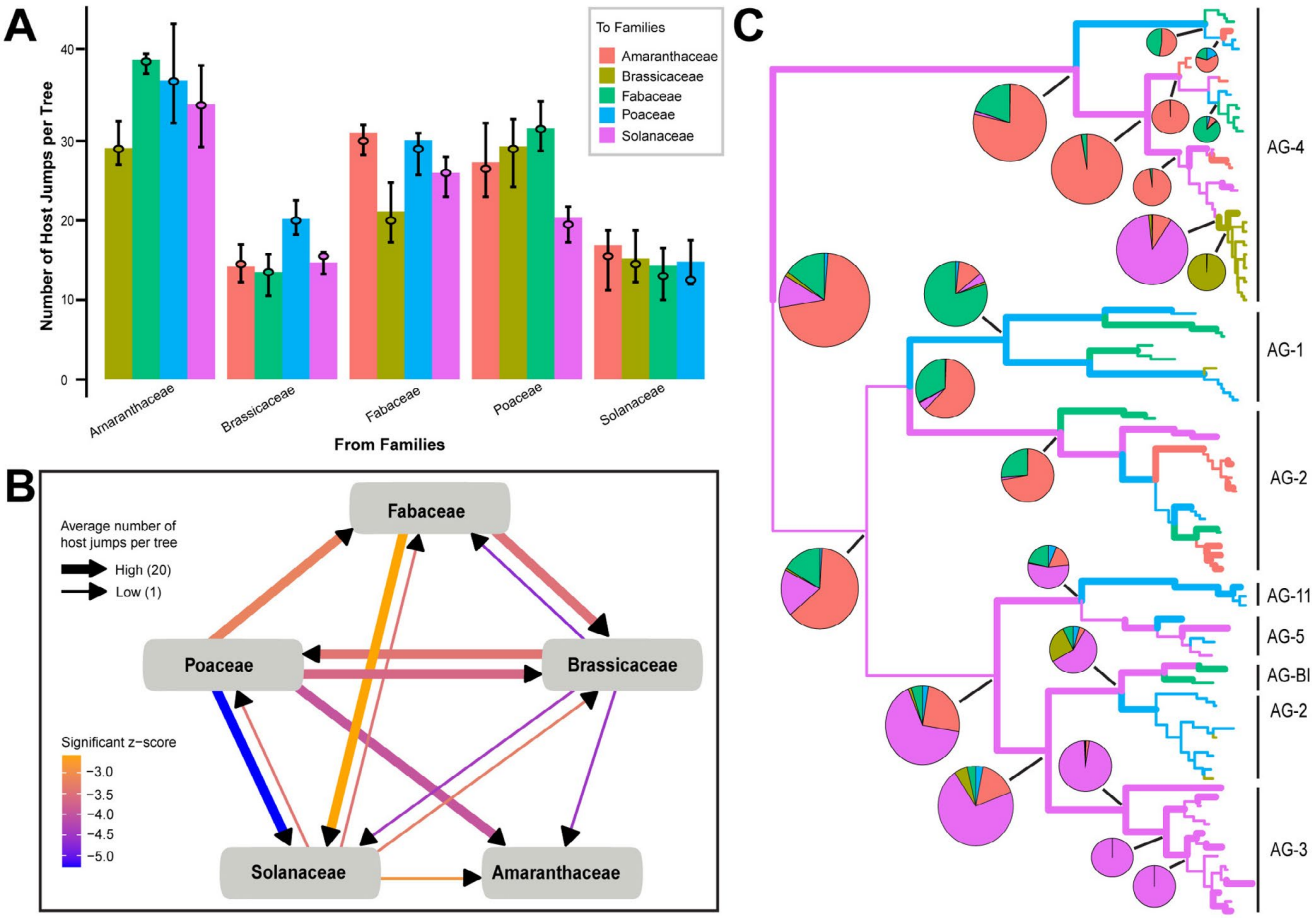
Inference of host‐switching events in *Rhizoctonia solani* estimated using Markov Jumps on BEAST v1.10.4. (A) Distributions of host jumps from one host family to another per tree across 10 host‐family‐stratified subsamples. Bars denote the mean host jump values per tree, the dots represent median values, and whiskers are standard error. (B) Network visualization of significantly constrained host jumps across major host families based on BEAST Markov‐jump models averaged over all 10 host‐family‐stratified subsamples. Line thickness represents the Markov‐jump count per tree averaged over all host‐family‐stratified subsamples. Line color reflects the statistical significance of those host‐switching events, with blue lines indicating more significant and constrained host jumps and lines of other colors reflecting significant but less constrained events. Only transitions with observed counts significantly lower than expected under the randomized model are shown. (C) Phylogeny of one of the host‐family‐stratified subsamples. Anastomosis groups (AGs) are labeled, and branches are colored according to the host‐families group from Panel A. Pie charts indicate the relative probability of host origin at the ancestral nodes. Line thickness corresponds to the posterior probability of the most likely host state.

We inferred the host‐switching events that may have occurred across the evolutionary history of *R. solani* by employing BEAST with Markov jumps. Convergence diagnostics indicated adequate MCMC sampling for the BEAST analyses, with reported key ESS values exceeding 200 (Table S4). To reduce imbalance representations of host groups in our results, we randomly subsampled an equal number of sequences from each main host group (*n* = 40), resulting in 200 sequences per subset. This approach was used because host‐family representation differed substantially among major reported AGs in the full ITS dataset, with some AG‐host‐family combinations more highly represented than others (Figure S3). The outgroup ( *A. rolfsii* ) was excluded because the primary focus of this analysis was on the ecological relevance of host‐switching events across the evolutionary history of *R. solani* . AG groups associated with Amaranthaceae had the highest number of jumps to other host families, with most exceeding 30 jumps per tree (Figure 3A). Upon comparing the empirical observation to the randomized host‐tip label model, none of the outgoing transitions from Amaranthaceae to other host families were greatly supported, while three other host families could transition into this host family, indicating that Amaranthaceae is a substantial recipient (Figure 3B). Poaceae, on the other hand, was identified as the major donor of host‐switching events in *R. solani* with a significantly high number of host jumps into all other host families. The most common recipients of jumps from Poaceae were Brassicaceae and Fabaceae, with a median of 32 jumps in each host family. Brassicaceae and Solanaceae had a relatively low number of host jumps per tree (Figure 3B). However, the network diagram showed that Brassicaceae and Solanaceae were still involved in inferred transitions to all other host families, namely Poaceae (*N*_Brassicaceae_ = 20) and Amaranthaceae (*N*_Solanaceae_ = 17). All statistically significant host‐switching events in our analyses had negative *z*‐scores, implying that the observed host‐switching events between major host families were less frequent than what would be anticipated by chance. We therefore refer to these patterns as significantly constrained host transitions. The greater the negative *z*‐score, the more limitation the host family experiences while transitioning to other host families. Fabaceae, with a *z*‐score between −3.5 and −2.5, exhibited constrained outgoing transition at a lesser extent, indicating that they occurred more often than Solanaceae (*z*‐score between −3.5 and −2.8), followed by Brassicaceae (*z*‐score between −4.7 and −3.4) and Poaceae (*z*‐score between −5.3 and −3.1).

The ancestral host‐state reconstruction of *R. solani* reveals contrasting host associations between AG‐3 and other AGs (Figure 3C), consistent with our observations in the ML tree (Figure 2A, Figure S1). The tree topology was likewise consistent, with nearly all major clades exhibiting posterior probabilities greater than 0.9, although several internal branches within AG‐4, AG‐2, and AG‐11 showed lower support values. All five host families were represented on the root node, with Amaranthaceae accounting for the most, Solanaceae and Fabaceae for the intermediate amount, and Brassicaceae and Poaceae for the least. Ancestral nodes of many AGs such as AG‐1, AG‐2, and AG‐4 exhibited dynamic shifts between multiple host groups, suggesting broader host ranges. Specifically, the ancestral node of AG‐4 was associated with Amaranthaceae, Fabaceae, and Solanaceae. Amaranthaceae and Fabaceae were the only host families associated with the ancestral nodes of the two subgroups that diverged from AG‐4. In one subgroup, both host groups were equally represented, while Amaranthaceae constituted the majority in the other subgroup. The latter grouping subsequently diverged into two subgroups that displayed distinct patterns of host association (Figure 3C). One subgroup was exclusively comprised of Amaranthaceae, whereas the other subgroup continued to be associated with both host groups. The first subgroup then reverted to a generalist state, interacting with all five host groups, while the latter subgroup expanded its host associations to include Brassicaceae, ultimately becoming predominantly associated with this family. Additionally, the most recent common ancestor (MRCA) shared by AG‐2 and AG‐3 showed diverse host group associations. The host range of this MRCA was dominated by Solanaceae, followed by Amaranthaceae, Brassicaceae, and equal proportions of Fabaceae and Poaceae. Following the divergence from the MRCA, the ancestral node of AG‐2 still maintained the multihost state, with the latter lineages exhibiting growing affinity for Brassicaceae, Poaceae, and Fabaceae (Figure 3C). In AG‐3, the probability of transitions into Solanaceae increased significantly, accounting for over 80% of the host association of its ancestral node. As it continued to diverge, the probability of host association of AG‐3 with Solanaceae kept increasing, eventually becoming exclusive in more recent lineages (Figure 3C).

### 3.4 Population Structure of *Rhizoctonia solani* Across Host and Geographic Groups

**FIGURE 4:**
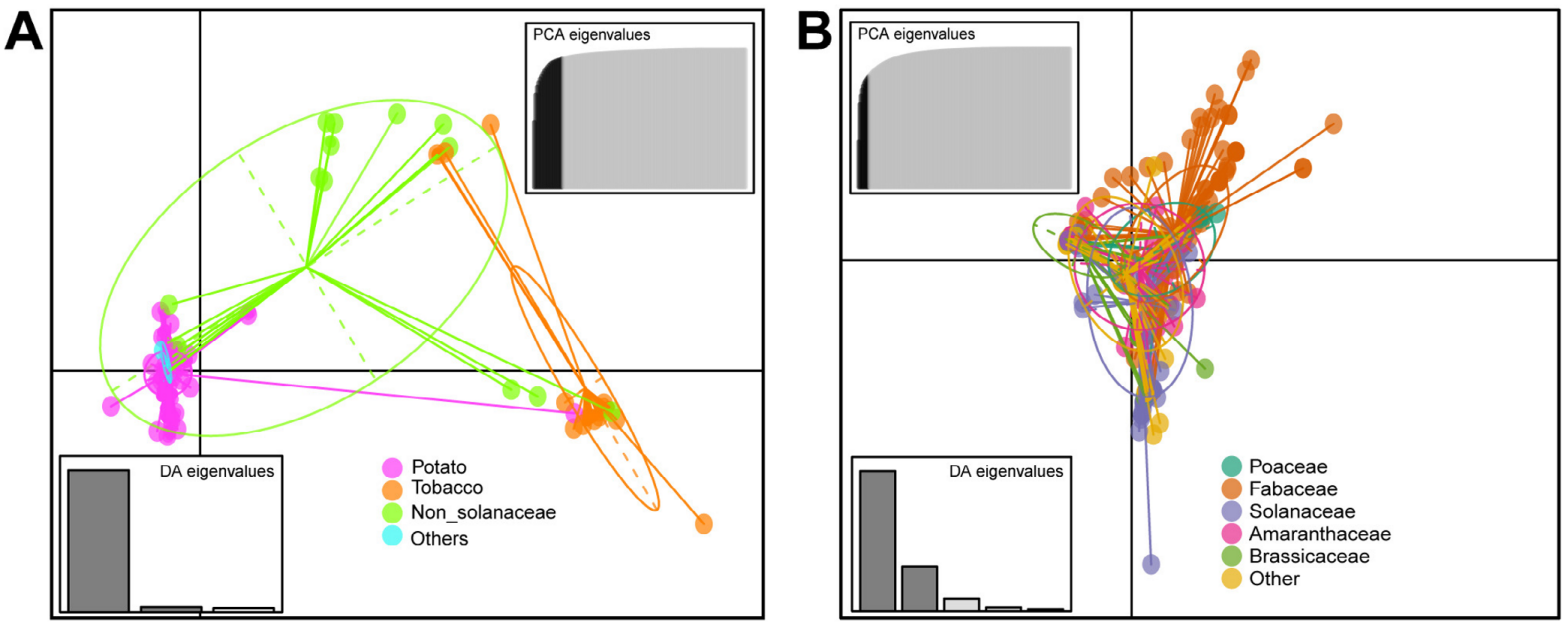
Discriminant analysis of principal component (DAPC) visualization of genetic structure in *Rhizoctonia solani* AG‐3 and AG‐4 in relation to reported host associations. DAPC was performed separately for the AG‐3 dataset (*n* = 914 sequences) and the AG‐4 dataset (*n* = 1174 sequences). (A) AG‐3 individuals are colored by reported host category: Potato, Tobacco, Others, and Non‐solanaceae. Tomato and black nightshade were grouped as ‘Others’ whereas canola, carrot, kiwifruit, lupin, rosemary, sand bluestem, soybean, and sugar beet were grouped as ‘Non‐solanaceae.’ (B) AG‐4 individuals are colored by reported host family. The ‘Other’ category included individuals from 26 less frequently represented host families, including Actinidiaceae, Apiaceae, Apocynaceae, Arecaceae, Asparagaceae, Asteraceae, Basellaceae, Bignoniaceae, Campanulaceae, Cannabaceae, Commelinaceae, Convolvulaceae, Cucurbitaceae, Ericaceae, Lamiaceae, Liliaceae, Linderniaceae, Malvaceae, Oleaceae, Pedaliaceae, Polygonaceae, Ranunculaceae, Rosaceae, Rubiaceae, Rutaceae, and Schisandraceae. Each point represents an individual sequence, and ellipses show dispersion of host‐associated groups in DAPC space. This host‐metadata grouped visualization was used to evaluate whether reported host categories (species or families) corresponded to genetic structure; independently inferred k‐means clusters and BIC results are shown in Figure S4.

Using the AG‐specific datasets, we employed DAPC to examine whether genetic structure in AG‐3 and AG‐4 corresponded more closely to host association or geographic origin. When individuals were analyzed at the continental scale, the BIC plots did not indicate strong geographic clustering, and DAPC scatter plots showed substantial overlap among geographic groups in both AG‐3 and AG‐4. In contrast, host association corresponded more closely to genetic structure, particularly in AG‐3. Unsupervised k‐means clustering based on the AG‐3 genetic data suggested approximately three genetic clusters according to the BIC curve (Figure S4). We then examined the correspondence between the inferred genetic clusters and reported host associations based on host metadata. Cluster 1 consisted primarily of potato‐associated genotypes (Figure 4A). It also included all individuals within the “Others” group and some individuals from the “Non‐solanaceae” group, although patterns involving the other‐Solanaceae group should be interpreted cautiously because of its small sample size. Cluster 2 consisted mainly of tobacco‐associated genotypes and was clearly distinctive from the potato‐associated cluster. Along with three non‐solanaceae genotypes (meaning individuals that were not isolated from solanaceous plants), one potato‐associated individual was also embedded in Cluster 2. Cluster 3 consisted primarily of non‐solanaceae genotypes, which showed greater dispersion in DAPC space and partial overlap with some tobacco‐associated individuals. For AG‐4, which has a broader reported host range, we similarly examined whether the inferred genetic structure corresponded to host‐family groups. The BIC curve of AG‐4 did not show a strong inflection point (Figure S4), suggesting the absence of a small number of well‐defined clusters. DAPC scatter plots showed some separation among individuals, but host‐family groups substantially overlapped and individuals from different host families frequently clustered together (Figure 4B). These results suggest that genetic structure in AG‐3 is strongly linked to host association, whereas AG‐4 shows weaker host‐associated genetic structure.

### 3.5 Population Structure and Genetic Diversity Patterns in *Rhizoctonia solani* AG‐3 and AG‐4

Since DAPC revealed clear genetic differentiation primarily associated with host group (Figure 4A) and only minimal clustering by geographic origin (Figure S5), we focused subsequent analyses on understanding the roles of host association in shaping the genetic diversity and structure of these *R. solani* populations. The alignment of the AG‐3 rDNA ITS dataset resulted in 958 aligned sites and 68 haplotypes. The population overall had a low haplotype diversity (*Hd*_Total_ = 0.269 ± 0.019) and moderate nucleotide diversity (*π*_Total_ = 0.005) (Table 1). The host species‐based population genetic analysis revealed that Potato had the most polymorphic segregating sites (*S*_Potato_ = 74) and mutations (Eta_Potato_ = 77), followed by Tobacco, which had 60 polymorphic sites and 78 mutations. Tobacco exhibited higher haplotype diversity (*Hd*_Tobacco_ = 0.989 ± 0.006) and nucleotide diversity (*π*_Tobacco_ = 0.016 ± 0.002) than Potato (*Hd*_Potato_ = 0.125 ± 0.016; *π*_Potato_ = 0.0004) and other host groups. All populations had negative Tajima's *D* scores, but only the Potato population had the statistically significant score. Similarly, Fu and Li's *F* statistic was also found negative and nonsignificant (*p* > 0.10) for Tobacco, others, and non‐solanaceae. These findings suggest a trend of population expansion across the AG‐3 population, with the effect being most pronounced in Potato.

**TABLE 1:** Population genetic parameters and neutrality tests for *Rhizoctonia solani* AG‐3 by host species.

Parameter	AG‐3 population				Total population
	By host species				
	Potato	Tobacco	Others^a^	Non‐solanaceae^b^	
No. of sequences	821	60	4	29	914
Gaps	442	311	320	371	456
Invariable sites	442	587	607	545	392
Polymorphism sites (*S*)	74	60	31	42	110
Total no. of mutations, Eta	77	78	32	45	117
No. of haplotypes (*H*)	49	49	4	15	68
Haplotype diversity (*Hd*)	0.125	0.989	1	0.924	0.269
	±0.016	±0.006	±0.177	±0.033	±0.019
Nucleotide diversity (*π*)	0.0004	0.016	0.025	0.016	0.005
		±0.002	±0.005	±0.003	
Tajima's *D*	−2.716***	−1.286	−0.862	−0.708	−2.350**
Fu and Li's *F*	−10.311*	−1.613	−0.822	−0.288	−8.862*

*Note:* Statistical significance: *p < 0.05, **p < 0.05, ***p < 0.001.

^a^ Individuals isolated from tomato and black nightshade.

^b^ Individuals isolated from canola, carrot, kiwifruit, lupin, rosemary, sand bluestem, soybean, and sugar beet.

The alignment of the AG‐4 rDNA ITS dataset resulted in 1286 aligned sites and 86 haplotypes. The host family‐based population genetic analysis revealed that *R. solani* sequences from Amaranthaceae had the most polymorphic segregating sites (*S*_Amaranthaceae_ = 228) and mutations (Eta_Amaranthaceae_ = 61) in the rDNA ITS region, followed by those from Solanaceae, which had 191 polymorphic sites and 212 mutations (Table 2). The haplotype diversity among AG‐4 was low (*Hd*_Total_ = 0.705 ± 0.01), with Amaranthaceae, Poaceae, and Solanaceae exhibiting relatively greater haplotype diversity (*Hd*_Amaranthaceae_ = 0.867 ± 0.011; *Hd*_Poaceae_ = 0.838 ± 0.045; *Hd*_Solanaceae_ = 0.715 ± 0.0009) than Brassicaceae (*Hd*_Brassicaceae_ = 0.224 ± 0.032). The overall nucleotide diversity was moderate (*π*_Total_ = 0.0178) across the AG‐4 population. Notably, Poaceae exhibited the highest nucleotide diversity (*π*_Poaceae_ = 0.0417 ± 0.001) in contrast to the lowest nucleotide diversity observed in Brassicaceae (*π*_Brassicaceae_ = 0.0061 ± 0.001). Many populations had significantly negative Tajima's *D* scores and Fu and Li's *F* statistics, except for Brassicaceae and Poaceae.

**TABLE 2:** Population genetic parameters and neutrality tests for *Rhizoctonia solani* AG‐4 by host families.

Parameter	AG‐4 population						Total population
	By host families						
	Amaranthaceae	Brassicaceae	Fabaceae	Other^a^	Poaceae	Solanaceae	
No. of sequences	300	290	245	112	47	180	1174
Gaps	909	805	1024	903	763	915	1070
Invariable sites	149	447	149	293	415	180	26
Polymorphism sites (*S*)	228	34	113	90	108	191	190
Total no. of mutations, Eta	269	35	141	97	121	212	313
No. of haplotypes (*H*)	61	12	30	14	21	24	86
Haplotype diversity (*Hd*)	0.867	0.224	0.639	0.534	0.838	0.715	0.705
	±0.01	±0.03	±0.03	±0.003	±0.05	±0.001	±0.01
Nucleotide diversity (π)	0.035	0.006	0.0205	0.013	0.042	0.017	0.0178
	±0.02	±0.01	±0.002	±0.003	±0.01	±0.003	
Tajima's *D*	−2.147**	−1.32	−2.374*	−2.40**	−0.73	−2.651***	−2.61***
Fu and Li′s *F*	−3.336*	−0.79	−5.754*	−4.279*	−2.31	−6.494*	−6.522*

*Note:* Statistical significance: *p < 0.05, **p < 0.01, ***p < 0.001.

^a^ Individuals isolated from 26 different host families, including Actinidiaceae, Apiaceae, Apocynaceae, Arecaceae, Asparagaceae, Asteraceae, Basellaceae, Bignoniaceae, Campanulaceae, Cannabaceae, Commelinaceae, Convolvulaceae, Cucurbitaceae, Ericaceae, Lamiaceae, Liliaceae, Linderniaceae, Malvaceae, Oleaceae, Pedaliaceae, Polygonaceae, Ranunculaceae, Rosaceae, Rubiaceae, Rutaceae, and Schisandraceae.

To further quantify host‐associated population differentiation, we performed AMOVA and pairwise distance‐based *F*_ST_ analyses among host‐defined populations within AG‐3 and AG‐4. AMOVA showed strong host‐associated structuring in AG‐3 (Table S5), with host‐defined population groups explaining 91.8% of ITS variation (ΦST = 0.918, *p* < 0.001). In contrast, only 6.4% of ITS variation in AG‐4 was explained by host‐defined grouping (ΦST = 0.064, *p* < 0.001). Pairwise *F*_ST_ analyses further supported this contrast (Table S6). In AG‐3, the differentiation between potato‐ and tobacco‐associated populations was significantly strong (*F*_ST_ = 0.702, *p* = 0.001), and the differentiation between tobacco‐associated and non‐Solanaceae populations was also significantly strong (*F*_ST_ = 0.570, *p* = 0.001). Potato‐associated populations showed moderate differentiation from non‐Solanaceae populations (*F*_ST_ = 0.232, *p* = 0.001). Comparisons involving other Solanaceae, such as tomato and black nightshade, labeled as “Others”, should be interpreted cautiously because this group only contained four sequences in our dataset. In AG‐4, pairwise differentiation was more heterogeneous and generally weaker, with *F*_ST_ values ranging from 0.003 to 0.378 across host‐family comparisons. The strongest differentiation was observed between Brassicaceae‐ and Fabaceae‐associated populations (*F*_ST_ = 0.378, *p* = 0.001) and between Brassicaceae‐ and Poaceae‐associated populations (*F*_ST_ = 0.348, *p* = 0.001).

### 3.6 Haplotype Structure of *Rhizoctonia solani* AG‐3 and AG‐4 Across Host Groups

**FIGURE 5:**
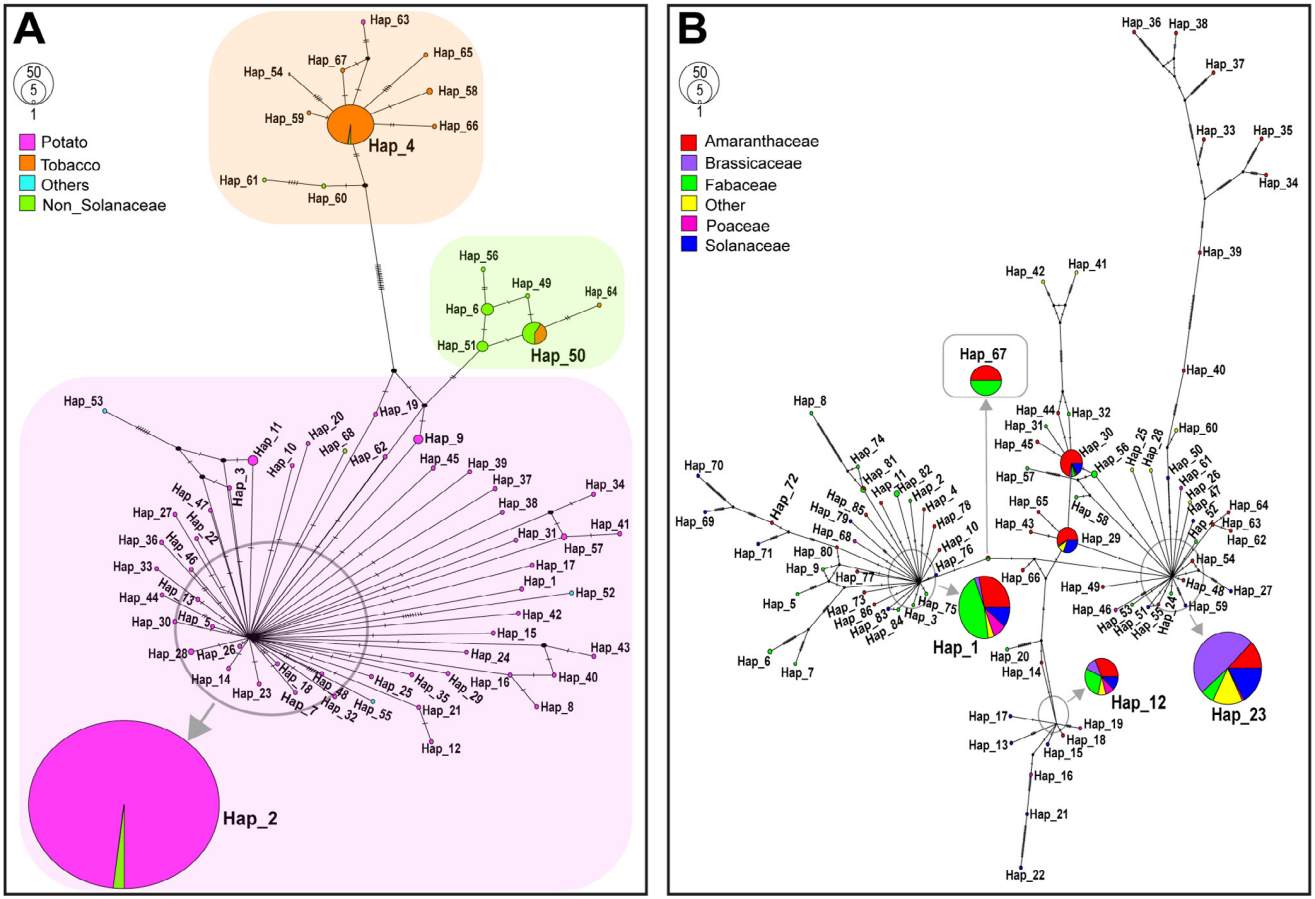
Haplotype network for the rDNA‐ITS sequences of *Rhizoctonia solani* populations. (A) The haplotype network illustrates genetic distance among *R. solani* isolates from four host groups (Potato, Tobacco, Others, and Non‐solanaceae) for the AG‐3 population. (B) Haplotype network for *R. solani* AG‐4 population from different host families. Each circle represents a unique haplotype, with the size proportional to the number of samples for that haplotype. Transverse lines represent a mutational step while black circles represent unsampled or extinct haplotypes.

The host‐based haplotype network supported previously observed genetic diversity in the AG‐3 population (Figure 5A). Two major haplotypes were observed, with 95.59% of them being exclusive to individual populations within these four host groups (Potato, Tobacco, others, non‐solanaceae). Hap_2 was positioned as the central haplotype of the “Potato cluster,” which consisted of *R. solani* AG‐3 isolates derived from potato hosts and served as a hub from which many other haplotypes diverged. The Potato cluster preserved a high degree of genetic similarity with limited but significant variation, as indicated by the short mutational steps separating Hap_2 from most minor haplotypes. The “Tobacco cluster,” which was composed of isolates derived from tobacco hosts, centered at Hap_4. The Tobacco cluster underwent a series of mutation steps and became genetically distinct from the Potato cluster, with no direct connections or shared haplotypes observed between the two. Nevertheless, the Tobacco cluster included Hap_63, a Potato haplotype that did not appear to be closely related to the major Potato cluster. The “non‐solanaceae cluster,” which consisted of isolates from canola, carrot, soybean, and other non‐solanaceae hosts as detailed in Table 1, centered at Hap_50 and showed greater affinity to the major Potato cluster than did the Tobacco cluster. Although a notable overlap of Tobacco haplotypes was observed within the non‐solanaceae cluster, these Tobacco haplotypes remained distinct from those in the Tobacco cluster.

The patterns of genetic differentiation by host family or group were less clear in AG‐4 compared to AG‐3. Five major haplotypes in AG‐4 were associated with multiple host families (Figure 5B), with significant overlap between host groups. The AG‐4 haplotype network also revealed unequal host associations across these major haplotypes. Although primarily associated with Brassicaceae, Hap_23 showed equal association with both Amaranthaceae and Solanaceae, with the least representation from Fabaceae, followed by Poaceae. Hap_1 had a greater representation from Amaranthaceae, Fabaceae, and Poaceae, while significantly underrepresenting Brassicaceae and Solanaceae in comparison to Hap_23. Hap_67, positioned between Hap_1 and Hap_23, was predominantly composed of Amaranthaceae and Fabaceae.

## 4 Discussion

Our study provides compelling evidence that ITS phylogenetic signal is broadly consistent with traditional *R. solani* anastomosis group (AG) classifications based on hyphal compatibility. Our analyses also revealed contrasting patterns of host‐associated genetic structure between *R. solani* AG‐3 and AG‐4 populations. Phylogenetic reconstruction, Markov‐jump inference of host‐transition patterns, and population structure analyses identified clear host‐associated clustering within AG‐3, whereas AG‐4 had weaker and more heterogeneous host‐associated structure. Our findings suggest that AG‐3 diverged from a polyphagous and multihost ancestor before becoming predominantly associated with Solanaceae, with evidence of further host‐associated specialization within this lineage. By comparison, AG‐4 retained a more generalist pattern of host association across multiple host families. The placement of most AGs in our ITS phylogeny corresponded with reported *R. solani* AG designations from publicly available databases, supporting the utility of ITS sequences for resolving broad population structure within the *R. solani* species complex. The largely congruent topologies of the ITS‐only and concatenated ITS+TEF‐1α phylogenies indicate that AG‐level placement was generally robust across single‐ and multilocus analyses. This correspondence between molecular phylogenetic signal and reported AG designations is supported by recent genome‐scale work showing that single‐copy genes can recover clustering consistent with reported fusion groups and AG‐3 subgroup designations (Xu et al. 2024). Our ITS marker‐based study complements that genome‐scale work by providing broader global sampling for examining AG‐level placement and host‐associated genetic structure across the *R. solani* species complex. When reconstructing the genetic relationships among these AGs, we observed that most recognized groups formed monophyletic clades supported by strong bootstrap values exceeding 70. Although AG‐4 appeared monophyletic in subset phylogenies, the basal node was weakly supported with a bootstrap value of 53. The full ITS phylogeny also revealed AG‐4 sequences being scattered across multiple clades. This pattern likely reflects the inclusion of genetically divergent subgroups within AG‐4, such as homogeneous group (HG)‐I, HG‐II, and HG‐III, which are known to vary in virulence and genetic identity (Kuninaga et al. 1997). This suggests that ITS is suitable for distinguishing AG‐level relationships but lacks the resolution to resolve finer‐scale divergence among subgroups within AGs. For instance, AG‐2 did not form a well‐supported monophyletic group in the ITS‐only phylogeny, as indicated by a basal node bootstrap value below 50. Instead, AG‐2 was divided into two well‐supported subgroups: one consistently clustered with AG‐BI in both the full and ITS‐only subset phylogenies, while the other grouped closely with AG‐5 across all phylogenies. This pattern is consistent with previous work proposing AG‐BI as a subgroup of AG‐2 based on strong anastomosis compatibility (Carling et al. 2002). Our phylogeny also supports earlier findings that AG‐2 is polyphyletic, with subgroups AG‐2‐1, −2‐2, and −2‐3 forming separate clades (Budge et al. 2009), and AG‐2‐1 isolates from potato and cabbage clustering apart (Gonzalez et al. 2001). We also observed occasional and unexpected clustering among strains from different AGs, which might reflect incomplete lineage sorting (ILS) or hybridization events (Stewart et al. 2014). While the ITS region exhibited weak temporal signal for capturing historical divergence and limited resolution for finer‐scale relationships, such as those among divergent subgroups within AG‐2, it nonetheless provided sufficient phylogenetic signal to differentiate distinct AGs and detect population‐level divergence.

As several distinct subpopulations of *R. solani* have been commonly reported on certain hosts or host families, *R. solani* provides a useful model system for exploring the roles of host diversity in the evolution of generalist fungal pathogens. Some *R. solani* AGs and their sublineages display clear host‐associated patterns, whereas others show inconsistent or ambiguous host associations. Our analyses suggest that *R. solani* exhibits primarily a generalist lifestyle, but that transitions among major host families are not random and are often significantly constrained. Within the inferred transition network, Poaceae, Brassicaceae, and Solanaceae appeared to act as reservoirs for the spread of *R. solani* to other host families. Among these host families, *R. solani* shows a greater tendency to switch from Poaceae to other host families than from Brassicaceae or Solanaceae. These patterns may be consistent with a coevolutionary arms race, wherein hosts may have evolved specific defense mechanisms that restrict the capacity of *R. solani* to switch. When *R. solani* became specialized to thrive on a particular host species belonging to Brassicaceae or Solanaceae, the pathogen's ability to colonize new or alternative host families may have been reduced. We also identified Amaranthaceae as an important recipient of inferred host‐switching events in *R. solani*. Amaranthaceae may represent a stable or frequently colonized host‐associated state within the transition network. One possible explanation for reduced transitions away from particular host states is that adaptation to a new host may involve trade‐offs that limit *R. solani* performance on alternative hosts, as predicted under antagonistic pleiotropy (Elena and Lenski 2003; Woolhouse et al. 2002). Moreover, the dynamics of host availability and competition are likely to exert selective pressure on *R. solani* (Kassen 2002; van Velzen and Etienne 2013), promoting adaptation to specific hosts only based on their abundance and the level of intraspecific competition.

Many AGs can switch between multiple host groups, highlighting their adaptive potential in response to host availability or environmental pressures. In AG‐4, the MRCA represented a generalist state, where its lineages were associated with multiple host groups. In the presence of other hosts during its evolutionary history, the group may have adapted to a specific host group and transitioned into a specialist state, as indicated by the dominance of Amaranthaceae at its intermediate nodes. Interestingly, our study suggests that the more recent lineages of AG‐4 may have regained broader host associations. Building on antagonistic pleiotropy theory, genetic traits that are associated with host specialization in certain AG‐4 subgroups may offer temporary adaptive benefits, but they may also limit host adaptability and incur substantial costs. When the drawbacks of specialization become greater than the advantages due to environmental or ecological changes, these groups may give up specialization and return to generalism. In contrast to the more dynamic host shifts inferred for AG‐4, AG‐3 shows a more restricted association with Solanaceae. Our results indicated that the MRCA shared by AG‐3 and other AGs was associated with a broader range of host groups before the AG‐3 lineage became predominantly associated with Solanaceae. We interpret this pattern as evidence of host‐associated specialization in AG‐3, but we cannot exclude the possibility that AG‐3 retains some capacity to colonize alternative hosts without a formal test of irreversibility. The persistence of Solanaceae‐associated AG‐3 lineages in the sampled dataset may reflect adaptation to this host family, although experimental studies would be needed to directly test host range and host‐specific fitness. These findings not only offer deeper insights into the evolutionary and phylogenetic context of the host‐specific patterns reported in a previous study (Keijer et al. 1997) but also suggest that host‐associated selection may have contributed to shaping the genetic structure of some *R. solani* populations or subpopulations.

Our population structure analyses further demonstrated that the genetic differentiation within AG‐3 population is strongly associated with host groups, resulting in two main clusters with varying levels of genetic diversity and a third partially overlapping cluster containing individuals that do not fit cleanly into either cluster. Together with the AMOVA and pairwise *F*_ST_ results, these findings support a strong association between host group and genetic subdivision within AG‐3. This pattern is consistent with the possibility that host‐associated selection has contributed to population subdivision within AG‐3, particularly in genetically uniform crop systems where repeated exposure to similar host genotypes can facilitate pathogen adaptation. Similar host‐associated patterns have been reported in other fungal pathogens, including the net blotch fungal pathogen *Pyrenophora teres* f. *teres* and the rice blast pathogen *Magnaporthe oryzae* during barley and rice domestication, respectively (Taliadoros et al. 2024; Couch et al. 2005).

In potato‐associated AG‐3, the dominance of a single haplotype, Hap_2 along with the low nucleotide and haplotype diversity observed in the ITS region may be consistent with evolutionary bottlenecks resulting from large‐scale monoculture of potato cultivation following potato domestication in the Andes region (Spooner et al. 2005). Given that the ITS is a multicopy ribosomal marker and that our dataset lacks sufficient temporal signal (Table S7), we cannot determine whether this pattern reflects a genome‐wide selective sweep or whether it coincides with potato domestication. Moreover, previous multilocus analyses reported high genetic diversity in potato‐associated AG‐3 populations (Ceresini et al. 2007), suggesting that the reduced ITS diversity observed here may not reflect genome‐wide diversity. Interpretation of diversity patterns in the ITS region should therefore be made with caution because ribosomal DNA repeats in many eukaryotic organisms may be influenced by concerted evolution and intragenomic variation (Ganley and Kobayashi 2007; Wang et al. 2023). Although concerted evolution is often expected in ribosomal repeats, non‐concerted evolution or region‐specific patterns have also been reported in some fungi, including within the 5S rRNA gene family (Rooney and Ward 2005). In *R. solani* , the ITS1 region is highly variable, whereas the ITS2 region is relatively conserved (Kuninaga et al. 1997), suggesting that different portions of the ITS region may evolve under different constraints. Similar patterns have been reported in *Ophiocordyceps sinensis* (Li et al. 2013), where different evolutionary forces acting across ITS regions were proposed to contribute to the coexistence of functional and non‐functional copies within the same genome. An alternative explanation is therefore that concerted evolution or other ITS‐specific processes may have homogenized ribosomal repeat variants within potato‐associated AG‐3 lineages. Rather than interpreting the reduced ITS diversity in potato‐associated AG‐3 as definitive evidence of a genome‐wide selective sweep, we interpret it as a marker‐specific pattern that may reflect demographic history, host‐associated selection, or ribosomal‐repeated evolution. Future studies using genome‐wide markers or whole‐genome sequencing will be needed to distinguish among these possibilities.

Within AG‐3 sub‐lineages, we also discovered that genetic differentiation was most evident between tobacco‐associated and potato‐associated populations, consistent with previous multilocus evidence that these host‐associated lineages represent distinct lineages with limited to no gene flow (Ceresini et al. 2007). Tobacco has been selected for resistance to some pathogens (McCorkle et al. 2013; Gillham et al. 1977), but breeding efforts for disease resistance have generally been less pronounced than those for potato, which has been extensively bred for resistance to *Phytophthora infestans* (Park et al. 2009; Haverkort et al. 2009) and potato cyst nematode (Gartner et al. 2021). Additionally, tobacco is widely cultivated with less intense monoculture practices, contributing to the relatively lower selective pressure on this tobacco‐associated AG‐3 population. Thus, we hypothesize that the tobacco‐associated AG‐3 population has retained greater genetic diversity without suffering from severe population size bottlenecks or random genetic drift (Lande 1976). While the evolution of the AG‐3 population is predominantly shaped by potato and tobacco, non‐Solanaceae host families such as Actinidiaceae, Amaranthaceae, and Apiaceae are present within these two distinct haplotype clusters. The overlap between these non‐Solanaceae host families and either potato or tobacco could indicate opportunistic host‐switching events, enabling evolutionary exploration in this Solanaceae‐adapted *R. solani* population through transient interactions with a wider range of hosts that share comparable environmental or ecological niches.

In contrast to the more host‐associated structure observed in AG‐3, AG‐4 tends to behave as an intermediate polyphagous fungus, exhibiting broader host compatibility and weaker host‐associated genetic differentiation. This flexibility suggests that AG‐4 can adapt to diverse ecological settings, showing significant plasticity in host adaptation. There are numerous overlaps across AG‐4 populations linked to different host families and even host species which is consistent with its elevated haplotype and nucleotide diversity across multiple host families. AG‐4 engages in regular expansions into new ecological niches by exploiting the availability of diverse hosts. Instead of exhibiting a clear clustering of haplotypes, the AG‐4 haplotype network exhibits a more highly interconnected structure, with numerous haplotypes associated with multiple host families. Notably, the existence of a more specialized connecting haplotype between two multihost major haplotypes suggests that AG‐4 has experienced transient host‐switching events. These transitions likely enable the AG‐4 population to be more effective at colonizing different hosts, allowing for adaptive evolution to a range of new or alternative hosts, thereby avoiding strict specialization to a single host family (Nyman 2010). Pathogenicity assays and multiomics studies will be needed to determine whether these genetic patterns correspond to functional differences in host compatibility and to identify the genetic factors associated with host adaptation (Zrenner et al. 2020; Mat Razali et al. 2021). Nevertheless, the weaker host‐associated differentiation and interconnected haplotype structure observed in AG‐4 suggest that host switching or transient host associations may contribute to host‐associated diversification in generalist *R. solani* populations.

## 5 Study Limitations

We acknowledge a few limitations in our study. First, our single‐gene approach for certain analyses might not accurately confirm evolutionary relationships among closely related subpopulations due to the high variability in the ITS1 region of *R. solani* (Kuninaga et al. 1997), the presence of different ITS regions within a single isolate (Pannecoucque and Höfte 2009; Grosch et al. 2007) and even intranuclear variability, as documented in other fungal species (DePriest 1993). However, we confirmed the placement of most AGs by analyzing a smaller subset of sequences concatenated from ITS and TEF‐1α, which yielded phylogenies consistent with those inferred from ITS alone. This concordance supports the reliability of ITS for resolving broad relationships among *R. solani* AGs, while also indicating that ITS should be interpreted cautiously for finer‐scale relationships among closely related subgroups. This caution is consistent with previous studies showing that ribosomal markers alone may provide insufficient phylogenetic information to confidently resolve infrafamilial relationships or the evolutionary history among genera in Ascomycetes and potentially most Basidiomycetes (Duong et al. 2006; Peláez et al. 2008; Tang et al. 2009). Second, although our host‐family‐stratified subsampling approach ensured equal representation of the five major host families in the Markov‐jump analyses, it did not fully eliminate ascertainment bias inherent to GenBank‐derived datasets. A diagnostic summary showed that some host‐family‐stratified subsets remained dominated by particular AGs, likely reflecting the overrepresentation of economically important or well‐studied AG‐host combinations (Table S8). Therefore, inferred Markov‐jump counts should be interpreted as host‐transition patterns within the available sequence dataset rather than as direct estimates of host‐switching frequencies in natural populations. Third, we were unable to estimate the precise timing of these ancestral host‐switching events because the full ITS dataset had limited temporal signal (Table S7). Although the concatenated ITS+TEF‐1α dataset showed stronger temporal signal, it contained far fewer sequences and therefore did not provide sufficient taxon coverage for robust global‐scale time‐calibrated analyses. Fourth, the lack of genome‐scale data suitable for global‐scale population genomic analyses remains a major limitation of our study. Although such analyses would provide stronger resolution of evolutionary history within *R. solani* , generating a globally representative genomic dataset would require a large, coordinated international effort involving extensive sampling across host taxa and geographic regions. Despite these limitations, the current global‐scale ITS dataset provides valuable insights into the *R. solani* species complex, largely due to its broad coverage of strain, host, and geographical diversity. This coverage has enabled us to identify broad and recurring host‐associated population patterns within *R. solani* .

## 6 Conclusion

Taken together, our comprehensive study demonstrates that phylogenetic signal from nuclear ribosomal DNA (ITS) is consistent with reported AG morphological classification of *R. solani* . Although ITS alone has important limitations for resolving host‐associated evolutionary patterns, it remains the most broadly available marker for *R. solani* on a global scale. Therefore, the global‐scale dataset assembled in our study represents one of the most comprehensive resources currently available for examining host‐related genetic structure across the *R. solani* species complex. Patterns recovered from this global‐scale dataset suggest that genetic structure in *R. solani* is more closely associated with host identity than with geographic distance. The coexistence of generalist and specialist populations within this species complex reflects divergent evolutionary trajectories in a soil‐borne pathogen, with some populations showing stronger host‐associated subdivision and others retaining broader and less clearly partitioned host associations. Patterns of phylogenetic clustering, host‐transition inference, and population structure of specialist populations like AG‐3 are consistent with host‐associated specialization, including differentiation among isolates associated with different Solanaceae hosts. In contrast, generalist populations like AG‐4 are more likely to experience occasional or transient host associations, which could help maintain genetic diversity and adaptability across a wide range of hosts and ecological contexts. These findings demonstrate that host and lifestyle transitions in *R. solani* can be inferred from genetic and ecological associated patterns. Building on this, future work incorporating genome‐scale data, pathogenicity assays, and broader sampling to compare generalist populations with specialist populations, or specialist‐leaning subgroups within generalist lineages, could help test the mechanisms underlying host range variation and host‐associated specialization. Such insights would facilitate epidemiological prediction and the development of more targeted and effective disease management strategies across a wider range of economically important crops.

## Author Contributions

**Xin Zhi Khoo:** conceptualization (equal), data curation (lead), formal analysis (lead), methodology (lead), resources (equal), software (equal), validation (lead), visualization (lead), writing – original draft (lead), writing – review and editing (equal). **Robert M. Harveson:** conceptualization (supporting), investigation (supporting), resources (supporting), writing – review and editing (supporting). **Teddy Garcia‐Aroca:** conceptualization (equal), data curation (supporting), formal analysis (supporting), funding acquisition (lead), methodology (supporting), project administration (lead), resources (lead), supervision (lead), validation (supporting), visualization (supporting), writing – review and editing (equal).

## Funding

This work was partially funded by the University of Nebraska‐Lincoln Agricultural Research Division (ARD) and the Institute of Agriculture and Natural Resources (IANR).

## Conflicts of Interest

The authors declare no conflicts of interest.

## Supporting information


**Table S1:** Metadata of the full ITS dataset containing 4119 *Rhizoctonia solani* isolates and one outgroup sequence, including newly sequenced *R. solani* isolates from Nebraska.
**Table S2:** Metadata of the concatenated dataset containing 162 *Rhizoctonia solani* isolates and one outgroup.
**Table S3:** Summary of ITS alignment trimming sensitivity analyses for *Rhizoctonia solani* AG‐level phylogenetic relationships.
**Table S4:** BEAST convergence diagnostics for combined post‐burn‐in logs across 10 host‐family‐stratified subsamples.
**Table S5:** AMOVA of host‐defined population structure within *Rhizoctonia solani* AG‐3 and AG‐4.
**Table S6:** Pairwise distance‐based *F*_ST_ among host‐defined populations within *Rhizoctonia solani* AG‐3 and AG‐4.
**Table S7:** Preliminary temporal analysis for different datasets of *Rhizoctonia solani*.
**Table S8:** Summary of dominant *Rhizoctonia solani* anastomosis groups (AGs) within each major host family across 10 host‐family‐stratified subsampled subsets.

**Figure S1:** Comprehensive phylogenetic analysis of *Rhizoctonia solani* anastomosis groups (AGs) from publicly available, published sequences and newly sequenced specimens.
**Figure S2:** Sensitivity of AG‐level phylogenetic relationships to ITS alignment trimming strategy.
**Figure S3:** Host‐family representation of major reported *Rhizoctonia solani* anastomosis groups (AGs) in the GenBank‐derived full ITS dataset.
**Figure S4:** Unsupervised k‐means clustering of genetic structure in *Rhizoctonia solani* AG‐3 and AG‐4.
**Figure S5:** DAPC visualization of *Rhizoctonia solani* AG‐3 and AG‐4 by geographic origin.

## Data Availability

Sequence data used in this study are available under the NCBI accession numbers listed in Tables S1 and S2. Custom scripts used for metadata extraction, phylogenetic tree visualization, host‐transition network analysis, host‐family‐stratified subsampling, and population genetic structure analyses are available at GitHub: https://github.com/xkhoo/R‐solani‐host‐population‐structure. Upon request from the corresponding author, all microbial isolates used in this study are available under a Material Transfer Agreement.
