# Supplementary material for: Global‐Scale Patterns of Host Transitions Suggest Contrasting Levels of Specialization Within Rhizoctonia solani
Source: Ecol Evol. 2026 Sep 1;16(9):e74254. doi: 10.1002/ece3.74254 (PMC13531243; doi:10.1002/ece3.74254)

Supporting information for "Global-scale patterns of host transitions suggest contrasting levels of specialization within *Rhizoctonia solani.*"

Khoo, Xin Zhi, Harveson, Robert M., & Garcia-Aroca, Teddy

This file contains the supplementary figures associated with the manuscript.

Contents

Supplementary Figure S1. Comprehensive phylogenetic analysis of *Rhizoctonia solani* anastomosis groups (AGs) from publicly available, published sequences and newly sequenced specimens.

Supplementary Figure S2. Sensitivity of AG-level phylogenetic relationships to ITS alignment trimming strategy.

Supplementary Figure S3. Host-family representation of major reported *Rhizoctonia solani* anastomosis groups (AGs) in the GenBank-derived full ITS dataset.

Supplementary Figure S4. Unsupervised k-means clustering of genetic structure in *Rhizoctonia solani* AG-3 and AG-4.

Supplementary Figure S5. DAPC visualization of *Rhizoctonia solani* AG-3 and AG-4 by geographic origin.

Supplementary Figure S1. Comprehensive phylogenetic analysis of *Rhizoctonia solani* anastomosis groups (AGs) from publicly available, published sequences and newly sequenced specimens. A). Maximum likelihood (ML) phylogenetic tree of the full ITS dataset encompassing all 4,120 sequences. The tree demonstrates the relationship among these sequences based on their reported anastomosis groups (AGs) on NCBI (I). The ML tree is further supplemented with additional layers representing host families (II), collection year (III), and geographic region (IV). These layers are color-coded to highlight the diversity of host families, collection time and their geographical distribution across different AG clades. **B-E). ML phylogenetic tree of the remaining four subsets containing 500 rDNA-ITS sequences.** The phylogenetic topologies presented in **Panels A-E** demonstrate consistent structural relationships across all analyses. Across the full dataset and subset analyses, major AG-level relationships were broadly consistent, supporting the robustness of the observed phylogenetic structure.

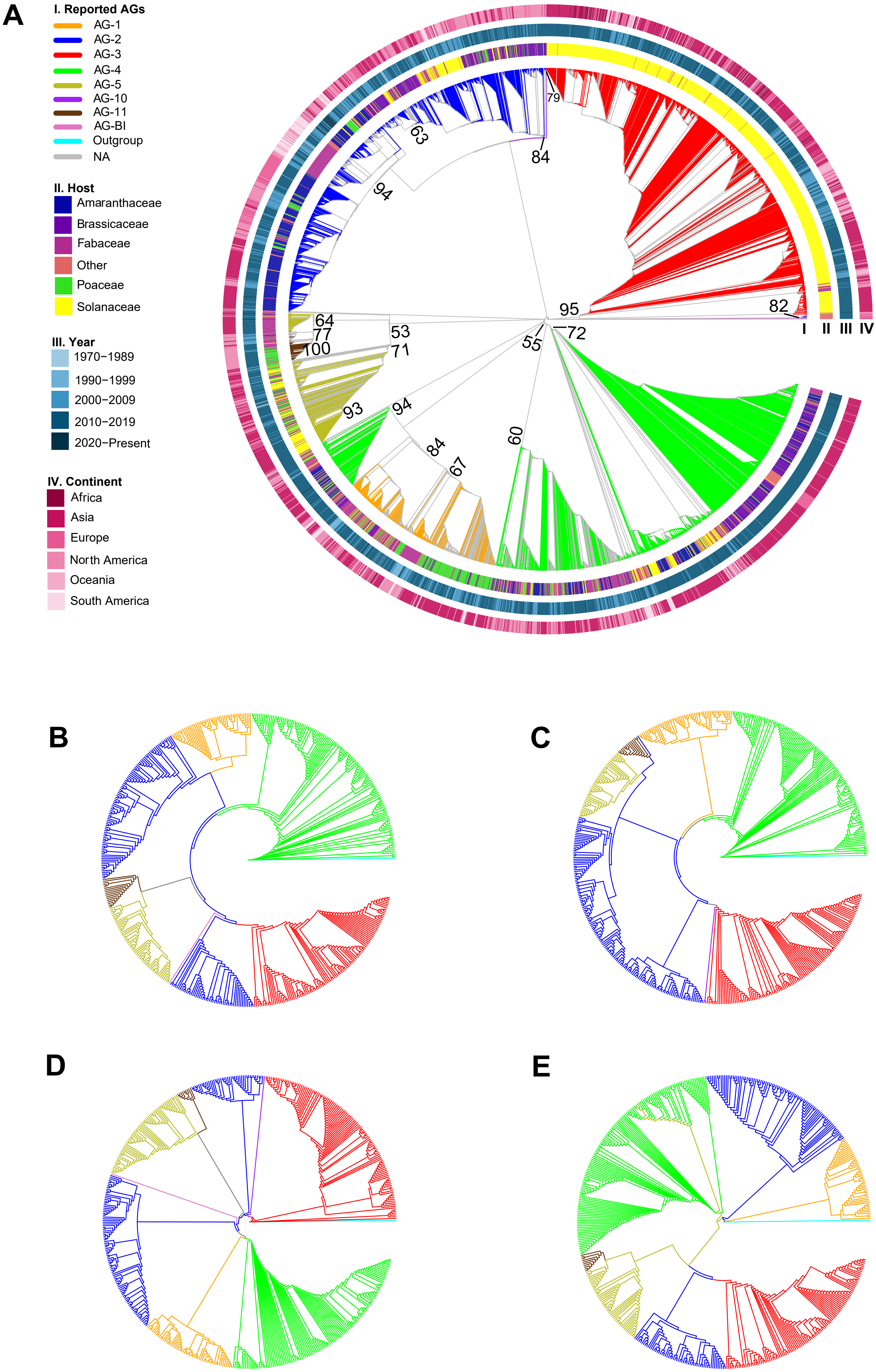
A
I. Reported AGs
AG-1
AG-2
AG-3
AG-4
AG-5
AG-10
AG-11
AG-BI
Outgroup
NA
II. Host
Amaranthaceae
Brassicaceae
Fabaceae
Other
Poaceae
Solanaceae
III. Year
1970−1989
1990−1999
2000−2009
2010−2019
2020−Present
IV. Continent
Africa
Asia
Europe
North America
Oceania
South America
I
II
III
IV
79
84
63
94
95
82
72
55
64
77
100
53
71
93
94
84
67
60
B
C
D
E

Supplementary Figure S2. Sensitivity of AG-level phylogenetic relationships to ITS alignment trimming strategy. Maximum-likelihood phylogenies were inferred from the full ITS dataset under different alignment trimming treatments: **A). untrimmed MAFFT alignment**, **B). ClipKIT gappy-mode alignment**, **C). ClipKIT kpic-mode alignment**, **D). ClipKIT smart-gap-mode alignment**, and **E). Gblocks-trimmed alignment.** The ClipKIT gappy-mode tree represents the primary trimming strategy used in the main analyses (Figure 2A) and is included here as a reference for comparison with the untrimmed and alternatively trimmed alignments. Major AG-level relationships were generally recovered across trimming strategies, indicating that the broad phylogenetic placement of *R. solani* AGs was robust to alignment trimming, although some bootstrap support values varied among trimming treatments.

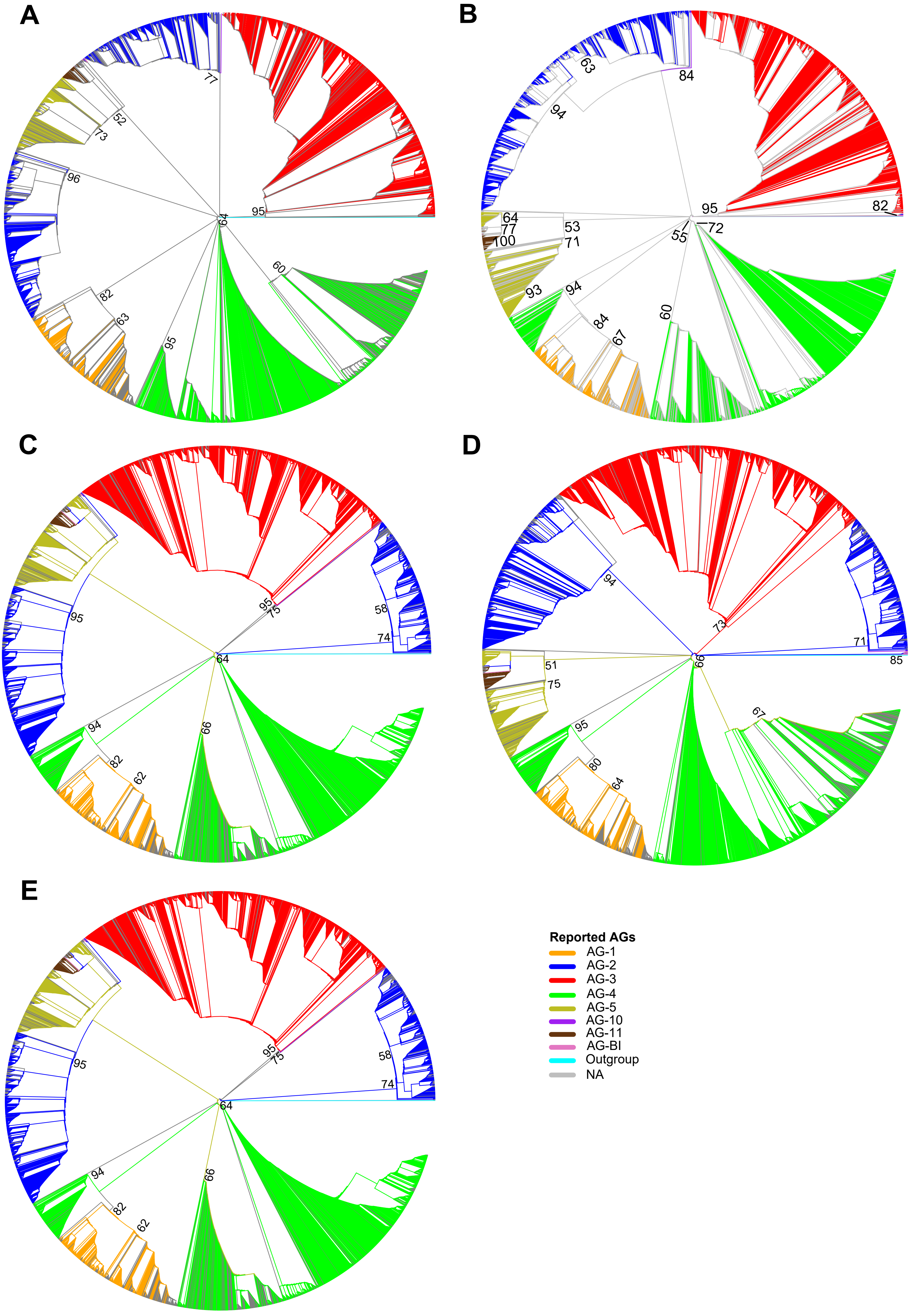
A
B
C
D
E
Reported AGs
AG-1
AG-2
AG-3
AG-4
AG-5
AG-10
AG-11
AG-BI
Outgroup
NA

Supplementary Figure S3. Host-family representation of major reported *Rhizoctonia solani* anastomosis groups (AGs) in the GenBank-derived full ITS dataset. Stacked bars show the percentage of sequences from each host-family category within major reported AGs. Each color represents a host-family category: Amaranthaceae, Brassicaceae, Fabaceae, Poaceae, and Solanaceae, or Other. The "Other" category includes 45 less frequently represented host families.

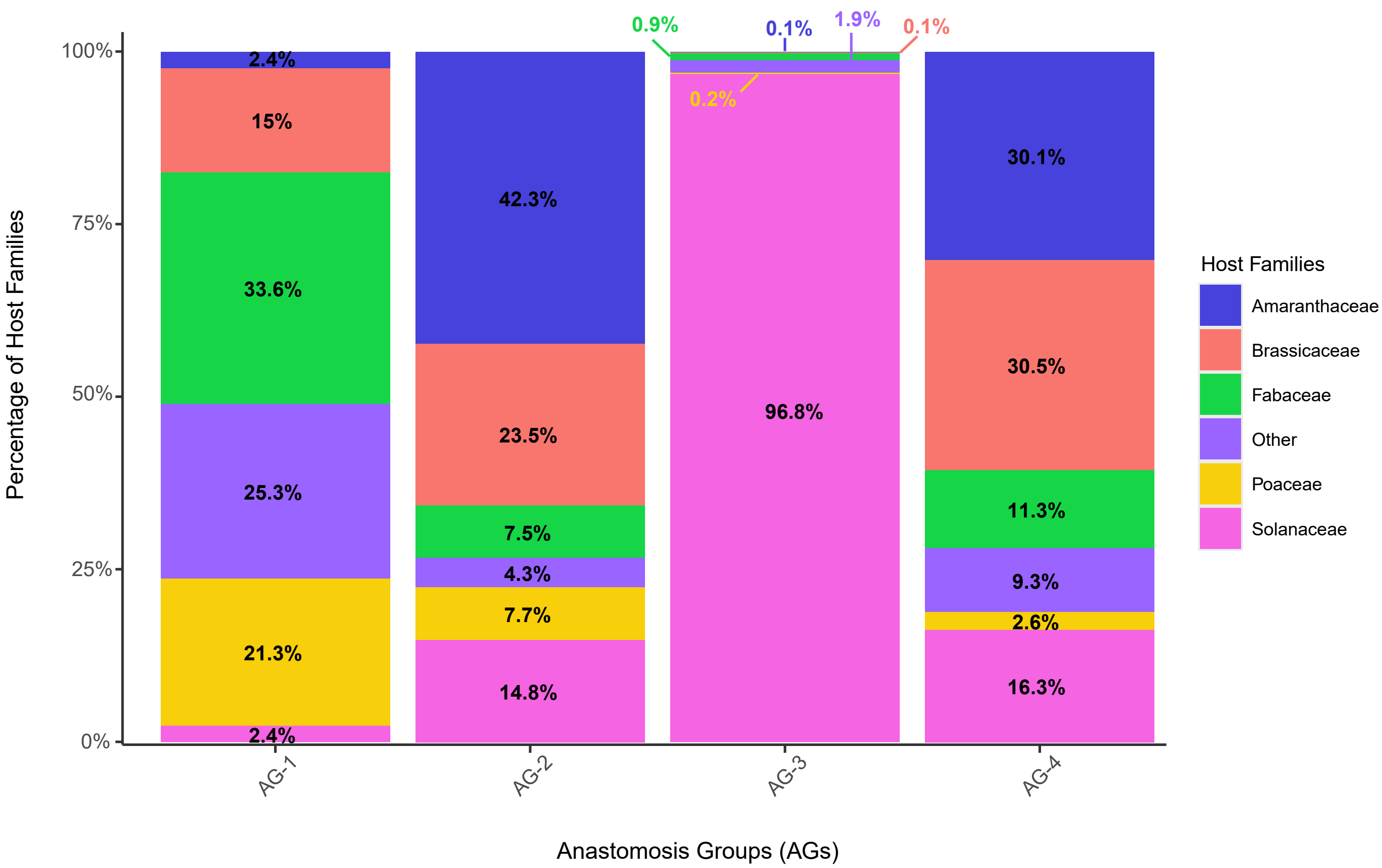

Supplementary Figure S4. Unsupervised k-means clustering of genetic structure in *Rhizoctonia solani* AG-3 and AG-4. The optimal number of genetic clusters was evaluated using Bayesian Information Criterion (BIC) values from k-means clustering for A). AG-3 and B). AG-4. Lower BIC values indicate better model fit, although the shape of the BIC curves was used to evaluate whether a clear clustering pattern was supported. **DAPC plots show individuals projected onto the first two discriminant functions for C). AG-3 and D). AG-4**, using genetic clusters inferred from k-means clustering rather than host or geographic labels.

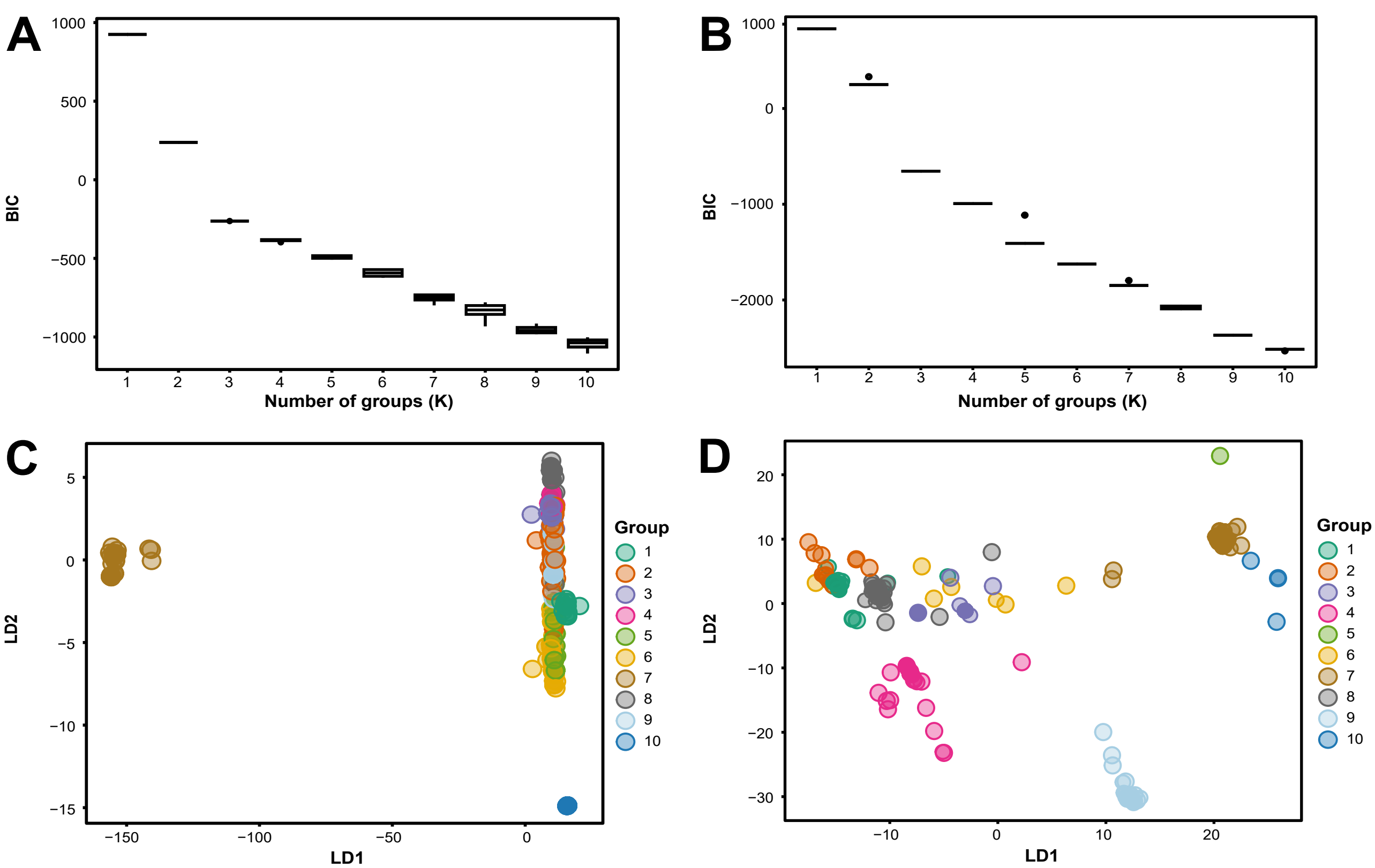

Supplementary Figure S5. DAPC visualization of *Rhizoctonia solani* AG-3 and AG-4 by geographic origin. Individuals were projected in DAPC space using continent of origin as the geographic grouping variable for A). AG-3 and B). AG-4. The AG-3 dataset included isolates from Africa, Asia, Europe, North America, Oceania, and South America, whereas the AG-4 dataset included isolates from Africa, Asia, Europe, North America, and South America. Substantial overlap among continental groups in both AG-3 and AG-4 suggests that geographic origin did not produce clearly separated genetic clusters in either AG-specific datasets.

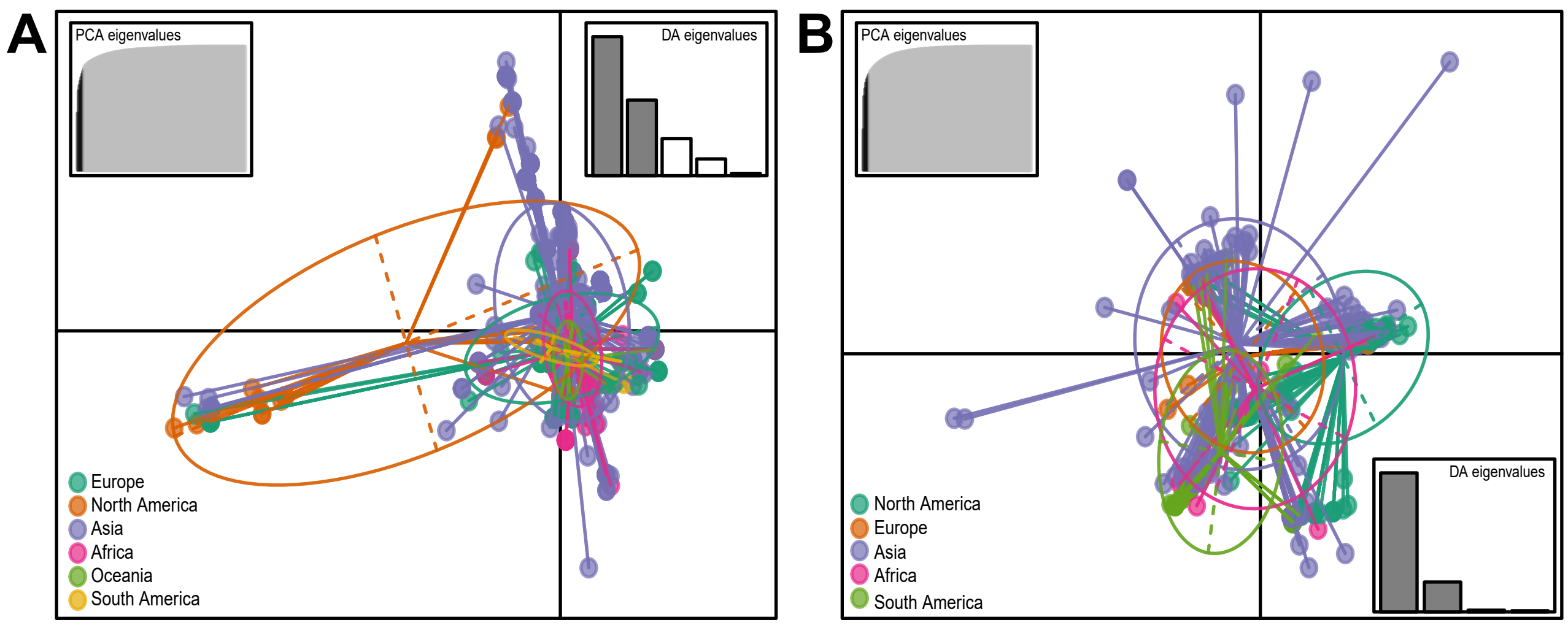

Supplement: Supplementary file 1 [file ECE3-16-e74254-s002.pdf]
